# Discovery of *N*′-(1-(coumarin-3-yl)ethylidene)benzenesulfonohydrazide as a novel wound healing enhancer: synthesis, biological assessment, and molecular modeling

**DOI:** 10.3389/fchem.2025.1621717

**Published:** 2025-08-08

**Authors:** Eman F. Khaleel, Heba Abdelmegeed, Abdel-Razik H. Abdel-Razik, Manal S. Ebaid, Ninh The Son, Nguyen Xuan Ha, Hoda Atef Abdelsattar Ibrahim, Mohamed A. Abdelrahman, Abdelsamed I. Elshamy, Jarosław Dziadek, Ahmed Sabt, Wagdy M. Eldehna

**Affiliations:** ^1^ Department of Medical Physiology, College of Medicine, King Khalid University, Asir, Saudi Arabia; ^2^ Chemistry of Natural Compounds Department, Pharmaceutical and Drug Industries Research Institute, National Research Centre, Cairo, Egypt; ^3^ Department of Histology, Faculty of Veterinary Medicine, Beni-Suef University, Beni-Suef, Egypt; ^4^ Department of Chemistry, College of Science, Northern Border University, Arar, Saudi Arabia; ^5^ Institute of Chemistry, Vietnam Academy of Science and Technology (VAST), Hanoi, Vietnam; ^6^ Institute of Natural Products Chemistry, VAST, Hanoi, Vietnam; ^7^ Pediatric Department, Faculty of Medicine, Cairo University, Cairo, Egypt; ^8^ Department of Pharmaceutical Chemistry, College of Pharmacy, University of Kut, Wasit, Iraq; ^9^ Department of Pharmaceutical Chemistry, Faculty of Pharmacy, Egyptian Russian University, Badr City, Cairo, Egypt; ^10^ Laboratory of Genetics and Physiology of Mycobacterium, Institute of Medical Biology of the Polish Academy of Sciences, Lodz, Poland; ^11^ Department of Pharmaceutical Chemistry, Faculty of Pharmacy, Kafrelsheikh University, Kafrelsheikh, Egypt

**Keywords:** biological evaluation, *in vivo* study, TNF-α, inflammatory mediators, sulfonamides

## Abstract

Wound healing poses a considerable challenge in the domain of medical science. In modern clinical practice, there is a growing trend towards using herbal compounds to aid in the repair process. Among these, coumarin, a phytochemical recognized for its antibacterial and wound-healing properties, has attracted significant interest. Consequently, the current research explores the potential benefits of employing coumarin to enhance wound healing in a murine model. The compound *N*′-(1-(7-hydroxy-2-oxo-2*H*-chromen-3-yl)ethylidene)-4-methylbenzene sulfonohydrazide (CBSH) was synthesized through the condensation of 7-hydroxy-3-acetyl coumarin with *p*-toluenesulfonylhydrazide and subsequently assessed for its antibacterial efficacy. CBSH showcased impressive antimicrobial prowess, demonstrating the values of minimum inhibitory concentration (MIC) 50, 40, and 40 μg/mL against the notorious *Staphylococcus aureus* MRSA, the resilient *Bacillus cereus*, and the formidable *Pseudomonas aeruginosa*. Subsequent *in vitro* and *in vivo* experiments were performed to assess its impact on the healing of skin wounds. The results indicated that CBSH significantly promotes the migration of skin fibroblast cells and enhances the wound healing process. Additionally, it facilitated the complete re-epithelialization of wounds. The formation of well-structured granulation tissue, along with a decrease in indicators of wound infection, is supported by histological analysis that demonstrates a minimal presence of inflammatory cells compared to untreated wounds. Furthermore, *in silico* molecular docking studies targeting key proteins involved in skin wound healing (COX-2, 5-LOX, COX-1, and TNF-α) demonstrated that COX-2 exhibited the highest binding affinity for CBSH, along with a stable complex during molecular dynamics simulations. Collectively, the results of this study indicate that CBSH may have a protective effect against infections in skin wounds, attributable to its antimicrobial properties.

## 1 Introduction

The skin functions as an essential organ in humans, fulfilling a vital role in preventing water loss, controlling bleeding, protecting against microbial invasion, and regulating body temperature (Ghaibi et al., 2015). Wound healing is a fascinating journey of restoration that unfolds in four unique stages: the initial stop of bleeding, the fiery onset of inflammation, the vibrant phase of proliferation, and the final touch of remodeling. During the inflammatory phase, the response is orchestrated by granulocytes and lymphocytes. The following phase of proliferation is marked by several key processes, including angiogenesis, epithelialization, the development and gathering of fibroblasts, and the production of collagen within the extracellular matrix (ECM). The final phase of wound healing, known as remodeling, involves fundamental alterations in the structure of collagen (Guo and DiPietro, 2010).

Wound healing is a multifaceted process that is affected by a variety of factors, including oxygen levels, infection presence, age, stress, nutrition, sex hormones, extracellular matrix, proteases, and cytokines (Ghaibi et al., 2015; Guo and DiPietro, 2010). Moreover, infections stand out as one of the leading culprits behind wound complications, thriving in environments that are just right for microorganisms to flourish (Bowler et al., 2001). The rise of multidrug-resistant infections adds another layer of complexity to creating innovative wound dressings that boast strong antimicrobial properties and promote significant healing (Ali et al., 2022). Also, a number of studies have delved into the antimicrobial prowess of natural compounds, like coumarin derivatives, targeting common wound bacteria, including *Staphylococcus aureus* and *Pseudomonas aeruginosa* (Martin et al., 2023; Ahmed et al., 2024). Thus, these remarkable compounds, with their antibacterial and antifungal capabilities, promise to accelerate and enhance the wound healing journey (Li et al., 2022).

**FIGURE 1:**
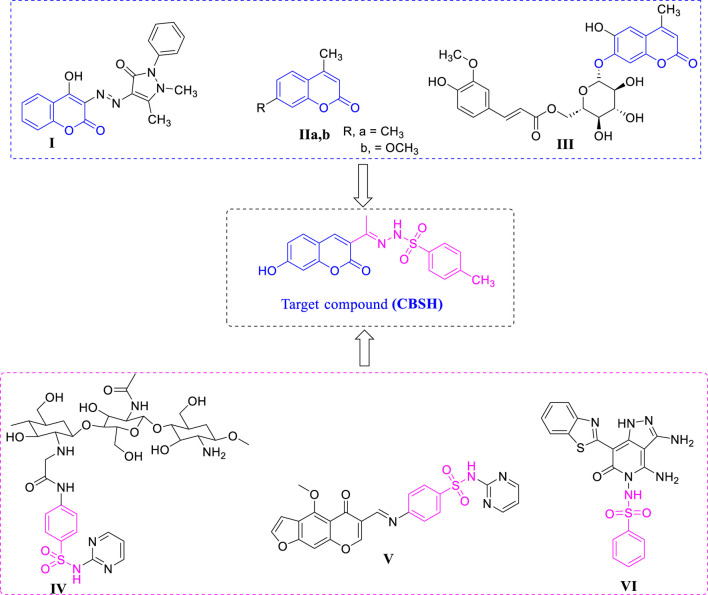
Structure for compounds that possess wound healing activity.

Coumarin, often referred to as benzopyran-2-one, belongs to a fascinating family of organic compounds that feature a delightful blend of aromatic rings. This intriguing class boasts a plethora of compounds found in nature, yet they can also be synthesized with ease, leading to a vibrant array of coumarin derivatives. These compounds have demonstrated remarkable pharmacotherapeutic potential, showcasing a wide range of biological activities (Annunziata et al., 2020). From acting as anti-leishmanial (Hassan et al., 2023; Gonçalves et al., 2020), anti-HIV (Batran et al., 2023; Sharapov et al., 2023), anticancer (Sabt et al., 2024a; Sabt et al., 2024b; Koley et al., 2024), antimycobacterial (Batran et al., 2024; Kassem et al., 2024), and antibacterial (Sabt et al., 2022; Karnaš et al., 2024). Coumarins are characterized by their considerable versatility, and their derivatives provide adaptable frameworks that exhibit a variety of biological activities, particularly in terms of antimicrobial properties that promote wound healing (Bettia et al., 2024; Afshar et al., 2020). For example, researchers J. Sahoo and P. Sudhir Kumar synthesized a series of 4-hydroxycoumarin derivatives conjugated with arylazo moieties. Their results indicated that most of these compounds displayed notable antimicrobial and wound-healing properties. Specifically, compound **I** (Figure 1) demonstrated significant efficacy, with a MIC of approximately 31.5, and exhibited a breaking strength (the force necessary to reopen a closed wound) that surpassed that of the control group (Sahoo and Kumar, 2015). FVA Dutra et al. documented the wound healing efficacy of two coumarin-derived compounds, specifically structures **IIa-b** (Figure 1), which possess significant potential for promoting wound repair (Dutra et al., 2024). Also, in 2024, Kim and colleagues successfully isolated six compounds from the root of *Nymphoides peltata* and assessed their efficacy in promoting wound healing. The findings indicated that coumarin glycoside **III** (Figure 1) exhibited the highest level of effectiveness among the compounds tested (Kim TY. et al., 2024).

Additionally, Schiff bases are a category of compounds distinguished by the existence of an azomethine group (-HC=N-), and they are recognized for their wide range of biological activities (Mushtaq et al., 2024). From a structural perspective, Schiff bases can be seen as nitrogen counterparts of aldehydes and ketones, where the carbonyl groups are replaced by azomethine or imino groups (Singh et al., 2021). Schiff bases are also important intermediates in organic syntheses and drug discovery (Carey and Sundberg, 2007; Makarem et al., 2019; Klika et al., 2022; Klika et al., 2021). Research has demonstrated that compounds featuring this structural motif exhibit various pharmacological properties, including antifungal, antibacterial, antiviral, and anti-tuberculosis effects (Sabt et al., 2023; Sabt et al., 2024c; Sabt et al., 2024d; Al-Warhi et al., 2024). Sulphonamides, which are chemically characterized by the presence of a sulfamoyl (–SO_2_NH–) group, are derivatives of amides. The first sulphonamide drug, prontosil, was discovered in 1932 and was utilized as an antibacterial agent (Durgun et al., 2020). Since then, sulphonamides have become among the most widely employed anti-infective agents globally. This class of compounds is of considerable biological significance due to their favorable oral absorption and renal excretion, which contribute to their relatively low toxicity, enhanced reactivity, and cost-effectiveness (De et al., 2018). Currently, sulphonamides are extensively utilized as inhibitors of carbonic anhydrase, anti-inflammatory agents, HIV protease inhibitors, and anticancer agents (Eldehna et al., 2024; Elsawi et al., 2023; Alafeefy et al., 2016; Zhao et al., 2012; Fares et al., 2017), in addition to their potent antimicrobial properties (Genç et al., 2008; Capasso and Supuran, 2015).

Sulphonamide Schiff base derivatives can be synthesized through the condensation of sulphonamide compounds containing at least one–NH_2_ group with aldehydes, resulting in the formation of biologically active compounds (Hirayama et al., 2002; Khan et al., 2015). In particular, Schiff base derivatives derived from sulfonamide drugs have garnered significant interest for their potential applications in wound healing (Hu et al., 2021; Elsayed et al., 2020). For instance, O.M. Dragostin et al. (2016) developed and synthesized novel antibacterial agents and enhanced wound dressings utilizing chitosan-sulfonamide derivatives. These derivatives exhibited markedly superior antibacterial efficacy compared to chitosan alone, with compound **IV** (Figure 1) showing exceptional activity against *Escherichia coli* ATCC 25922 and *S*. *aureus* ATCC 25922, with MIC values of 0.03 mg/mL and 1.25 mg/mL, respectively. Furthermore, this compound underwent additional *in vivo* assessments for wound healing, revealing a significant improvement in healing outcomes and enhanced epithelialization. In 2024, H.M. Abo-Salem and colleagues (Abo-Salem et al., 2024) published findings on the antimicrobial properties of a novel series of chromone and furochromone-based sulfonamide Schiff’s bases, identifying structure **V** (Figure 1) as the most effective compound. This compound was encapsulated in chitosan nanoparticles, and its efficacy in protecting skin wounds was assessed through *in vivo* studies. The results demonstrated complete wound re-epithelialization and epidermal hyperplasia. Also, compound **VI** (Figure 1) exhibited enhanced antibacterial, anti-inflammatory, and wound-healing properties when combined with nanofiber cellulose acetate/poly (lactic acid) (Elsayed et al., 2020).

To improve the effectiveness of dressing mats in combating bacterial infections and to facilitate the wound healing process, a novel compound, referred to as CBSH, has been synthesized. This compound incorporates coumarin, sulphonamide, and Schiff base moieties, and its antibacterial properties and potential applications in wound healing have been evaluated, as depicted in Figure 1. Furthermore, molecular docking analysis has provided valuable insights into the mechanisms of action, clarifying the interactions between CBSH and its target proteins. This interdisciplinary approach merges theoretical chemistry with pharmacological assessment, enabling a thorough investigation of the properties and prospective applications of these compounds in the field of drug discovery and development.

## 2 Results and discussion

### 2.1 Chemistry

**SCHEME 1:**
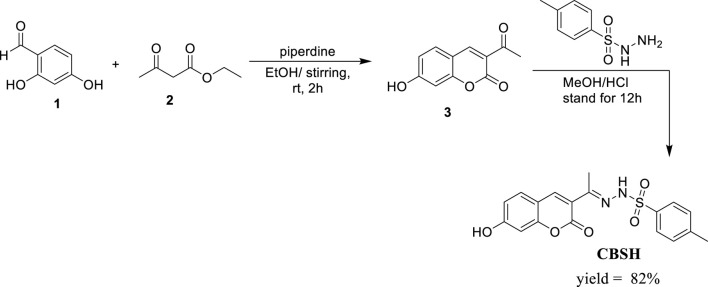
Synthesis of CBSH.

Preparation of the target compound CBSH is delineated in Scheme 1, which also illustrates the precursor, 3-acetyl-7-hydroxy-2*H*-chromen-2-one (3). A literature review indicates that numerous synthetic methodologies exist for the preparation of 3-acetyl-2*H*-chromen-2-one derivatives (Hassan et al., 2023). In the present study, we employed a Knoevenagel condensation reaction, which involved the room-temperature stirring of 2,4-dihydroxybenzaldehyde (1) with ethyl acetoacetate (2) in the presence of a catalytic quantity of piperidine in ethanol. This reaction yielded the compound 3-acetyl-7-hydroxy-2H-chromen-2-one (3). Subsequently, this compound was subjected to overnight stirring with *p*-toluenesulfonyl hydrazine in methanol. By incorporating a few drops of hydrochloric acid, the desired compound CBSH is successfully produced.

### 2.2 Biological assessments

#### 2.2.1 Antimicrobial activity assessed through *in vitro* methods

The potential of the newly synthesized compound to act as an antimicrobial agent can efficiently improve wound healing, since bacterial infection can hinder wound healing (Guo and DiPietro, 2010; Larouche et al., 2018). Hence, we utilized the agar cup plate diffusion technique to evaluate the antimicrobial potential of CBSH. In this respect, Methicillin-resistant *Staphylococcus aureus* (MRSA) (Gram-positive bacteria), *Bacillus cereus* (Gram-positive bacteria), and Carbapenem-resistant *Pseudomonas aeruginosa* (CRPA) (Gram-negative bacteria), along with fungal pathogens such as *Candida albicans* and *Aspergillus niger,* were investigated in the presence of CBSH and compound **3** (Supplementary Material).

As shown in Table 1, the parent compound **3** did not show any antibacterial activity against *S. aureus* (MRSA) and *P. aeruginosa* (CRPA) while showing moderate antibacterial activity against *B. cereus.* On the other hand, CBSH displayed moderate antibacterial activity against both Gram-positive and Gram-negative bacteria, with the highest antibacterial activity against *B. cereus*. Additionally, CBSH did not display antifungal activity toward *C. albicans* and *A. niger*. Moreover, the MIC of 3 and CBSH was determined as displayed in Table 2. CBSH compound showed an MIC of 50 µg/mL (MIC of compound **I** was 31.25) toward *S. aureus* (MRSA), which is lower than that of cephradine, polymyxin B, and ampicillin MIC values (>100 µg/mL, 80 µg/mL, >100 µg/mL, respectively), indicating its high potency.

**TABLE 1:** Antibacterial activity of CBSH using agar cup plate diffusion method.

Compound	Inhibition zone (mm)		
	*Staphylococcus aureus* MRSA	*Bacillus cereus*	*Pseudomonas aeruginosa*
3	ND	3	ND
CBSH	2	4	3
Ciprofloxacin^a^	5	6	4
Ampicillin^a^	ND^b^	3	ND^b^
Polymyxin B^a^	ND^b^	2	3
Kanamycin^a^	3	3	ND

^a^ Ciprofloxacin, Ampicillin, Polymyxin B, and Kanamycin were utilized as the standard practice as antibacterial agents at 20 µg/mL.

^b^ ND: not determined.

**TABLE 2:** Minimum inhibitory concentration (MIC) of CBSH.

Compound	MIC (µg/mL)		
	*Staphylococcus aureus* MRSA	*Bacillus cereus*	*Pseudomonas aeruginosa*
3	>100	80	>100
CBSH	50	40	40
Ciprofloxacin	10	5	10
Cephradine	>100	80	>100
Polymyxin B	80	30	20
Kanamycin	40	80	>100

On the other hand, ciprofloxacin and kanamycin had lower MIC values than CBSH (10 µg/mL and 40 µg/mL, respectively). In the case of *B. cereus,* CBSH had a MIC value of 40 µg/mL, which was lower than cephradine, ampicillin, and kanamycin MIC values (80 µg/mL, >100 µg/mL, 80 µg/mL, respectively), which indicates that compound CBSH is more potent than cephradine, ampicillin, and kanamycin. Furthermore, ciprofloxacin and polymyxin B had MIC values of 5 µg/mL and 30 µg/mL, respectively, which shows that CBSH was less potent than ciprofloxacin and polymyxin B against *B. cereus.* CBSH showed a MIC value of 40 µg/mL against *P. aeruginosa* (CRPA), which is less than cephradine, ampicillin, and kanamycin (MIC >100 µg/mL), which demonstrates that CBSH has higher activity than cephradine, ampicillin, and kanamycin. Meanwhile, ciprofloxacin and polymyxin B had MIC values of 10 µg/mL and 20 µg/mL, respectively, showing that both are more active than CBSH against *P. aeruginosa* (CRPA).

When comparing the antibacterial activity of CBSH and 3, we realize that the antibacterial activity of CBSH is superior to that of the parent compound 3. CBSH displayed potent antibacterial activity against *S. aureus* (MRSA), *B. cereus,* and *P. aeruginosa* (CRPA) with low MIC values (50 µg/mL, 40 µg/mL, and 40 µg/mL, respectively). Conversely, the parent compound 3 demonstrated very low antibacterial activity with high MIC values (>100 µg/mL, 80 µg/mL, >100 µg/mL, respectively). Collectively, these findings display that the antibacterial activity of CBSH is superior to that of the parent molecule **3** cephradine, ampicillin, and kanamycin, and less than that of ciprofloxacin, demonstrating the promising activity of CBSH as an antibacterial agent. Our data are in alignment with other studies that showed antibacterial activity of several coumarin derivatives (El-Sawy et al., 2024; Ranjan Sahoo et al., 2021; Cheke et al., 2022). The antibacterial activity of CBSH can be attributed to the presence of the coumarin nucleus, which displays antibacterial and antibiofilm activity against different bacteria, including *E. coli* (Lee et al., 2014)*, Salmonella Typhimurium* (Thakur et al., 2020), and *P. aeruginosa* (Qais et al., 2021). Collectively, the newly synthesized CBSH is a prominent antibacterial derivative that can be used to protect wounds from infection, leading to improved wound healing.

#### 2.2.2 *In vitro* evaluation of CBSH effect on cell migration

**FIGURE 2:**
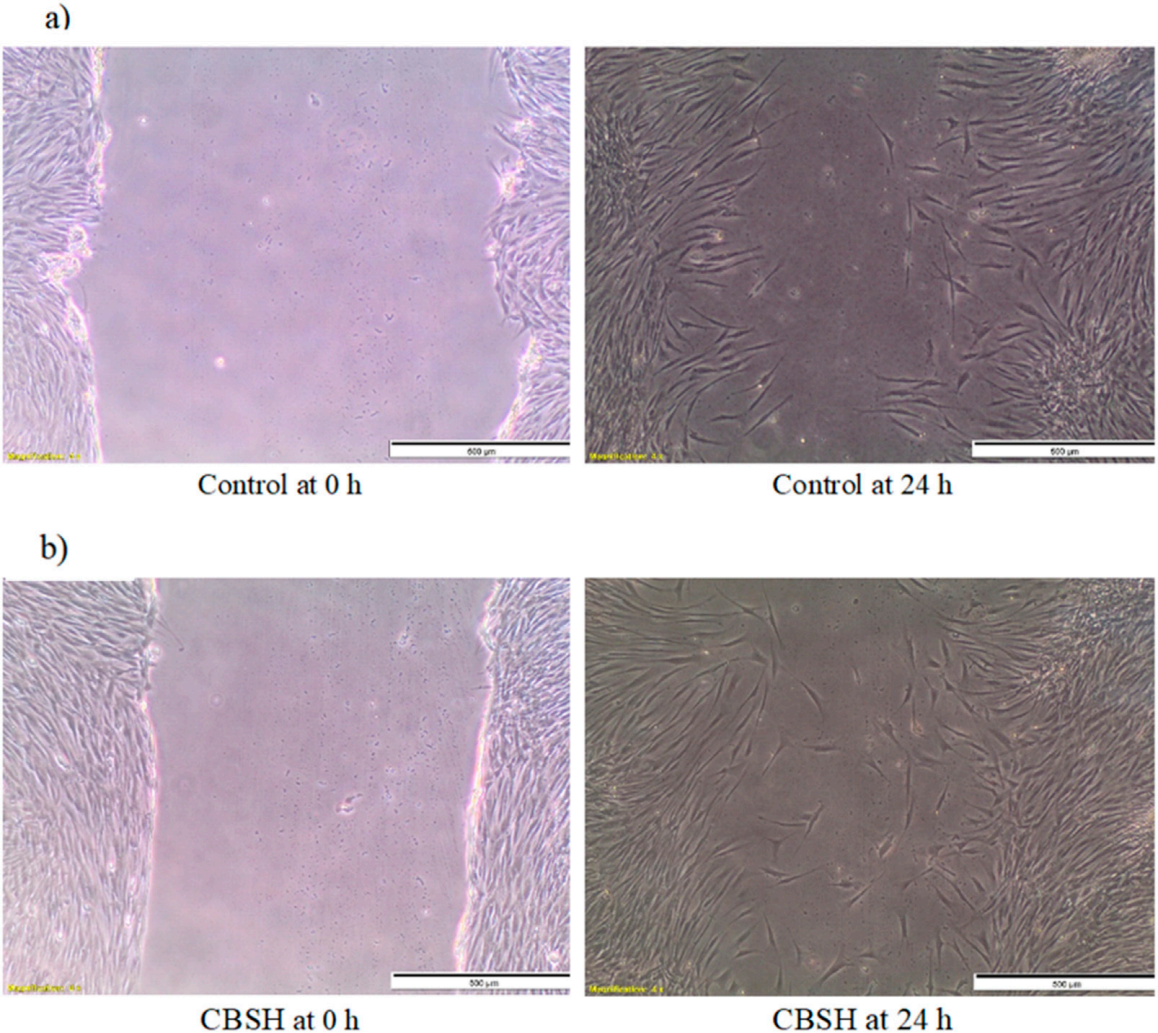
*In vitro* scratch wound assay for evaluation of cell migration for **(a)** non-treated cells and **(b)** CBSH-treated cells.

The effect of CBSH on human fibroblast cells was identified through scratch healing assessment using the *in vitro* method. A monolayer of BJ cells was created, a vertical scratch was made, and the cells were treated with CBSH for 24 h. As illustrated in Figure 2, the cells exposed to CBSH displayed increased cell migration, leading to scratch closure by 82.5% compared with non-treated control cells, which demonstrated wound closure of 71%. These results displayed that CBSH enhanced skin fibroblast cell migration and promoted wound healing. Several studies showed similar results where coumarin derivatives induced cell migration *in vitro*. Kim et al., demonstrated that peltatamarin A (6-hydroxy-coumarin-7-*O*-(6′-*O*-feruloyl)-*β-*D-glucopyranoside), which is a coumarin glycoside, promoted cell migration in the HaCaT cell scratch test (Kim T.-Y. et al., 2024). Geranyloxycoumarin, which is another coumarin derivative, displayed the ability to significantly induce collagen I expression when added to human skin fibroblasts *in vitro* (Adams et al., 2016). Similarly, umbelliferone (7-hydroxycoumarin) induced collagen I and fibronectin expression. It increased cell migration via increasing the synthesis of proteins involved in cell migration, such as the focal adhesion protein (EVL) and Fascin-1 (Kim D. Y. et al., 2024). These data align with our results, which highlight the promising effects of coumarin derivatives as wound healing boosters.

#### 2.2.3 *In vivo* assessment of CBSH effect on wound healing

Wound healing is a multifaceted process that can be enhanced through several coumarin derivatives and sulfonamide compounds (Afshar et al., 2020; Abo-Salem et al., 2024). As shown previously, CBSH demonstrated higher antimicrobial activity than ampicillin, kanamycin, and cephradine. Additionally, it induced wound healing in human fibroblast cells *in vitro*. Therefore, we assessed the potential of CBSH to induce wound healing *in vivo* by topically applying our novel compound to assess the impact of skin wounds in rats and monitor the subsequent effects on the wound-healing process. Incisions were made on the dorsal region of two groups, each consisting of five rats that either received no treatment or CBSH. The wound-healing process was monitored in each group over a duration of 2 weeks. The injuries of each group were carefully monitored and documented through photography on the day of the operation (day 0) and subsequently on days 3, 5, 8, 10, and 14 post-operation in order to assess the degree of wound closure.

**FIGURE 3:**
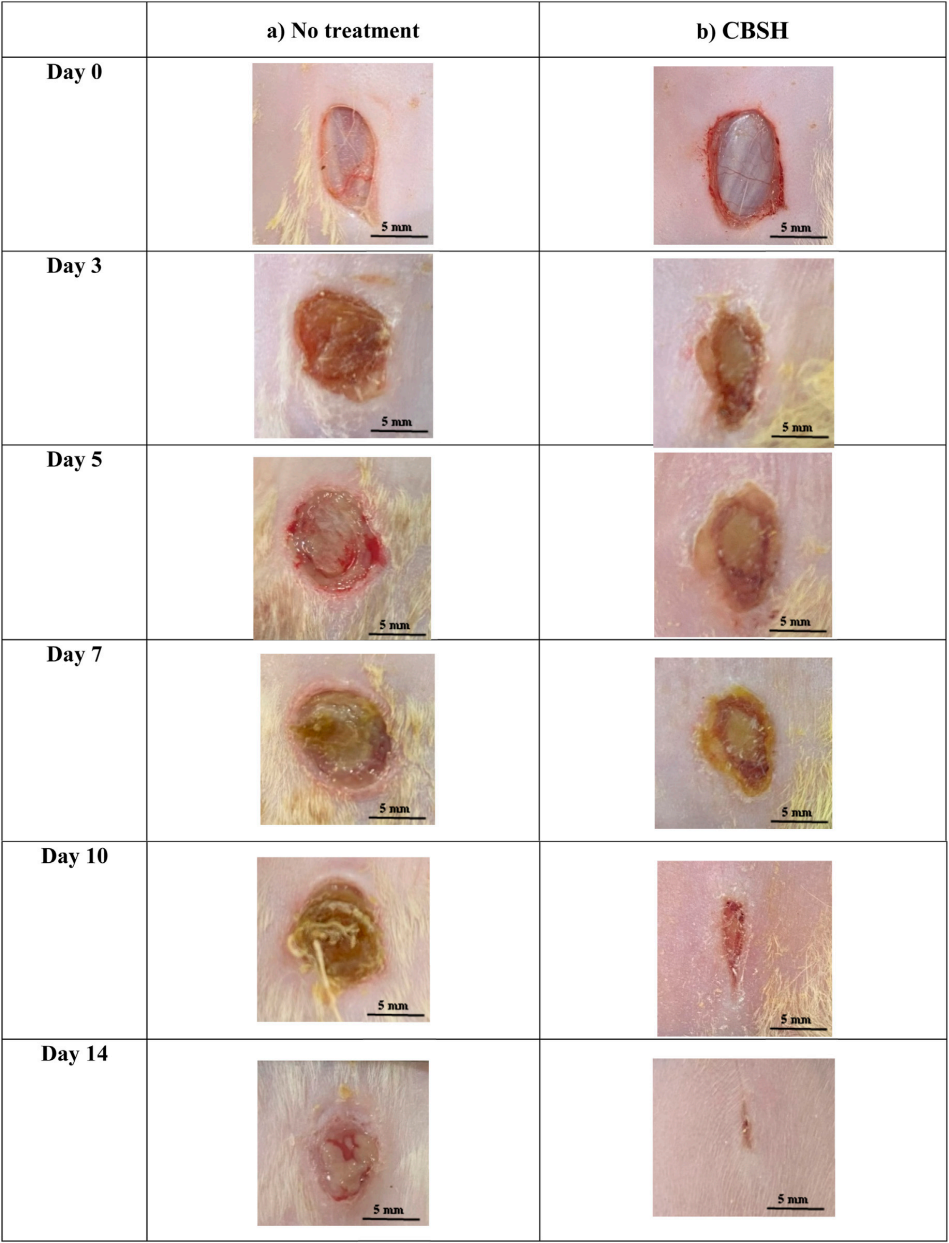
Illustrative images depicting the wound regions of the two groups (n = 5) showing the healing process: **(a)** No treatment, **(b)** CBSH as documented during a two-week timeframe.

**FIGURE 4:**
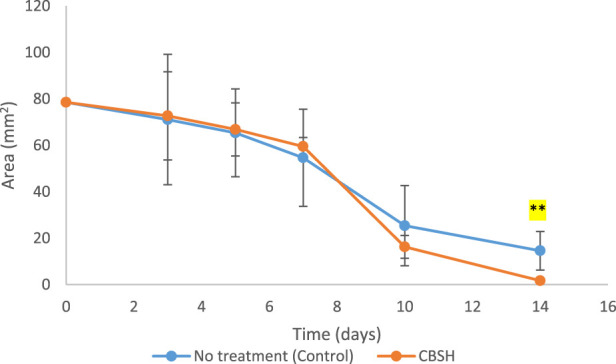
The alterations in wound size for both the untreated group and the group treated with CBSH were assessed over a span of 2 weeks (p-value <0.01).

As displayed in Figure 3, on day 14, The untreated group demonstrated insufficient advancement of the newly formed epidermis toward the wound’s center, which hindered complete wound closure. In contrast, by the 14th day, treatment with our newly synthesized derivative led to almost a complete wound closure (97.8%) and the smallest wound area compared with the non-treated group. CBSH treatment significantly enhanced the wound healing process by the 14th day (p-value < 0.01). This expedited wound-healing process led to a reduction in the size of the wound, as shown in Figure 4.

The data collectively demonstrate the substantial impact of CBSH in facilitating wound dressing and enhancing the process of wound closure *in vivo*. Our findings are consistent with other studies that showed other coumarins with similar ability to improve wound healing *in vivo*. Coumarin led to enhanced wound healing when applied topically to Balb/c mice. It improved re-epithelialization during the proliferative phase of wound healing (IRANJD, 2025). When certain coumarins are complexed with *β*-cyclodextrin to improve their solubility, they accelerate re-epithelialization and collagen deposition. The improved wound healing effect was demonstrated *in vivo* after topical application of 4,7-dimethyl-2*H*-chromen-2-one (DMC) and 7-methoxy-4-methyl-2*H*-chromen-2-one (MMC) in Swiss mice (Dutra et al., 2024).

An additional *in vivo* study developed new solid lipid nanoparticles with co-encapsulation of coumarin and Q10. The newly synthesized nanoparticles induced wound healing compared with both the control group and Mebo cream (Beta-sitosterol)-treated group (Alrubaye et al., 2025). Another study showed that using a cream containing phellopterin, a furanocoumarin, accelerated wound healing in diabetic mice. Such an effect was attributed to phellopterin induction of sirtuin 1 (SIRT1) and re-epithelialization promotion (Zou et al., 2022). Furthermore, umbelliferone was able to accelerate diabetic wound healing via activating and inducing the release of angiogenic mediators in the wound area (Efficacy of Topical Application of). Altogether, CBSH, along with many coumarin derivatives, have great potential to be used for accelerating wound healing *in vivo*.

#### 2.2.4 Histopathological assessment of wound healing

**FIGURE 5:**
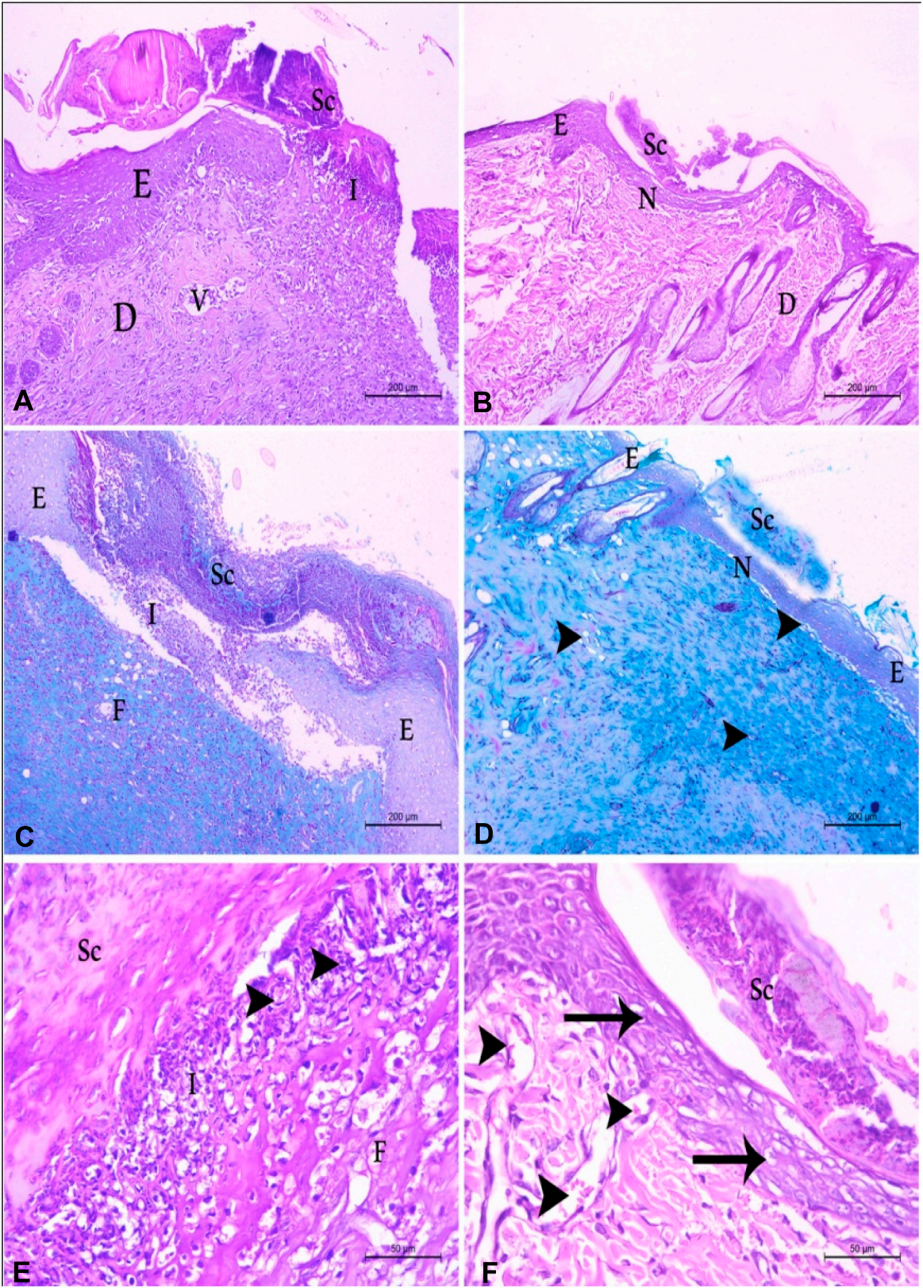
The histopathological picture of albino rat’s skin in control positive and CBSH groups **(A)** control positive group showing severe destruction in the epidermal (E) and dermal layers (D) with total loss of the epithelial covering. Firmly attached scar tissue is located above the destructed area (Sc). Sever inflammatory response in the affected area (I). (H&E Stain X100) **(B)** CBSH treated group showing complete epithelization of the wound area with underlying normal dermal tissue (N). Note, normal epidermal and dermal tissue on both sides of the wound area. (H&E Stain X100) **(C)** control positive group showing firmly attached scar tissue with collagen fibers is located above the destructed area (Sc), inflammatory cells in the affected area (I) and massive fibrosis (F) in the dermis of the affected area. (Masson’s trichrome Stain X100) **(D)** CBSH-treated group showing normal skin structure in the wound area (N), covered with loosely attached fine scare with fine collagen fibers, underlying normal dermal tissue with newly formed blood vessels (arrowhead). (Masson’s trichrome Stain X100) **(E)** a higher magnification of the wound area in the control positive group showing scar tissue (Sc) and inflammatory cells (I) with micro-abscess formation (arrowhead). (H&E Stain X400) **(F)** a higher magnification of the wound area in CBSH treated group showing complete epithelization of the wound area (arrow) covered by loosely scar (Sc) with underlying dermal tissue with new vascular tissue formation (arrowhead) (H&E Stain X400).

Histopathologically, the control positive group showed severe destruction and loss of the epidermal layers. The wound area is covered by firmly attached scar tissue with collagen fibers. The underlying dermal layers possessed massive fibrosis and severe inflammatory response in the wound area with marked congestion of blood vessels and massive infiltration with neutrophils and lymphocytes in addition to the appearance of micro-abscess in the wound area (Figures 5A,C,E). On the contrary, the use of the compound CBSH induces complete epithelization of the wound area, covered with a loosely attached fine scar with fine collagen fibers. The underlying dermal tissue appeared with abundant fibrous collagen fibers with newly formed blood vessels (Figures 5B,D,F). Using CBSH on a wound topically ameliorates the wound healing.

### 2.3 Molecular docking and dynamics

*In silico* screening, studies were performed to elucidate the inhibitory potential of compound CBSH using reverse docking techniques. We have identified key proteins that play an essential role in the process of skin wound dressing and conducted an analysis of their binding interactions, focusing on the active sites of the selected enzymes. The strongest binding affinities were identified as the optimal conformations of the protein-ligand complexes. The docking findings revealed that COX-2 displayed the highest binding affinity with CBSH (−8.706 kcal/mol), surpassing other proteins such as TNF-α (−8.506 kcal/mol), 5-LOX (−8.127 kcal/mol), and COX-1 (−7.53 kcal/mol).

**FIGURE 6:**
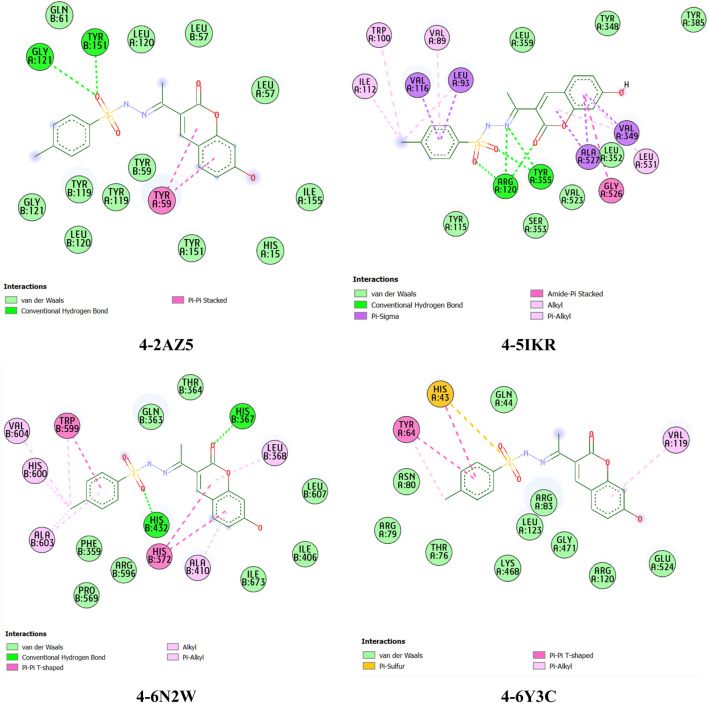
2D interaction view of CBSH within the active site gorge of proteins TNF-α (PDB code: 2AZ5), 5-LOX (PDB code: 6N2W), COX-1 (PDB code: 6Y3C), and COX-2 (PDB code: 5IKR).

Our findings highlight CBSH as a promising potential inhibitor of cyclooxygenase-2 (COX-2), making it an attractive molecular target for tissue regeneration. Notably, this compound forms a more stable complex with COX-2 compared to COX-1, as indicated by a significant energy difference of approximately 1.176 kcal/mol. Detailed interaction analyses revealed that CBSH establishes a hydrogen bond with Arg120 and Tyr355 in COX-2, along with specific hydrophobic interactions (Figure 6). These include alkyl and pi-alkyl interactions with Trp100, Ile112, Val89, and Leu531, pi-sigma interactions with Val116, Leu93, Ala527, and Val349, and an amide-pi stacked interaction with Gly526. Interestingly, pi-alkyl interactions with Ala527 and Val349 were also observed in the co-crystalized complex of the well-known COX-2 inhibitor rofecoxib, further supporting the relevance of these residues in stabilizing ligand binding at the COX-2 active site (Pham et al., 2023).

Compound CBSH also demonstrated the ability to form stable complexes with other targets through multiple interactions with critical amino acid residues. For instance, it creates hydrogen bonds with Gly121 in chain A and Tyr151 in chain B of TNF-α, similar to the positive control SPD304 (Ha and Le, 2024). Furthermore, the compound interacts with 5-LOX by forming hydrogen bonds with His367 and His432, alkyl and pi-alkyl interactions with Ala410, Leu368, Val604, His600, and Ala603, and a pi-pi T-shaped interaction with His372. Notably, residues Trp599 and His372 play a vital role in the active site of 5-LOX (Gilbert et al., 2020). As shown in Figure 6, COX-1 residues exhibit fewer interactions with CBSH compared to COX-2, underscoring its potential selectivity for the two cyclooxygenase enzymes. On the other hand, mefenamic acid, which is complexed with Cox-2 (−7.23 kcal/mol), showed thirteen π-alkyl interactions with Val349, Leu531, Val523, Val116, Tyr355, Ala527, and Leu352. Additionally, two hydrogen bonds were observed with Ser530 and Tyr385 (Supplementary Material).

To further validate the stability of the COX-2– CBSH complex, MD simulations were performed, with RMSD values monitored throughout the simulation. RMSD analysis provides insight into the structural deviations of atoms (protein and ligand) from their reference conformation, where higher RMSD values suggest greater instability and structural alterations (Liu and Kokubo, 2017; Schreiner et al., 2012).

**FIGURE 7:**
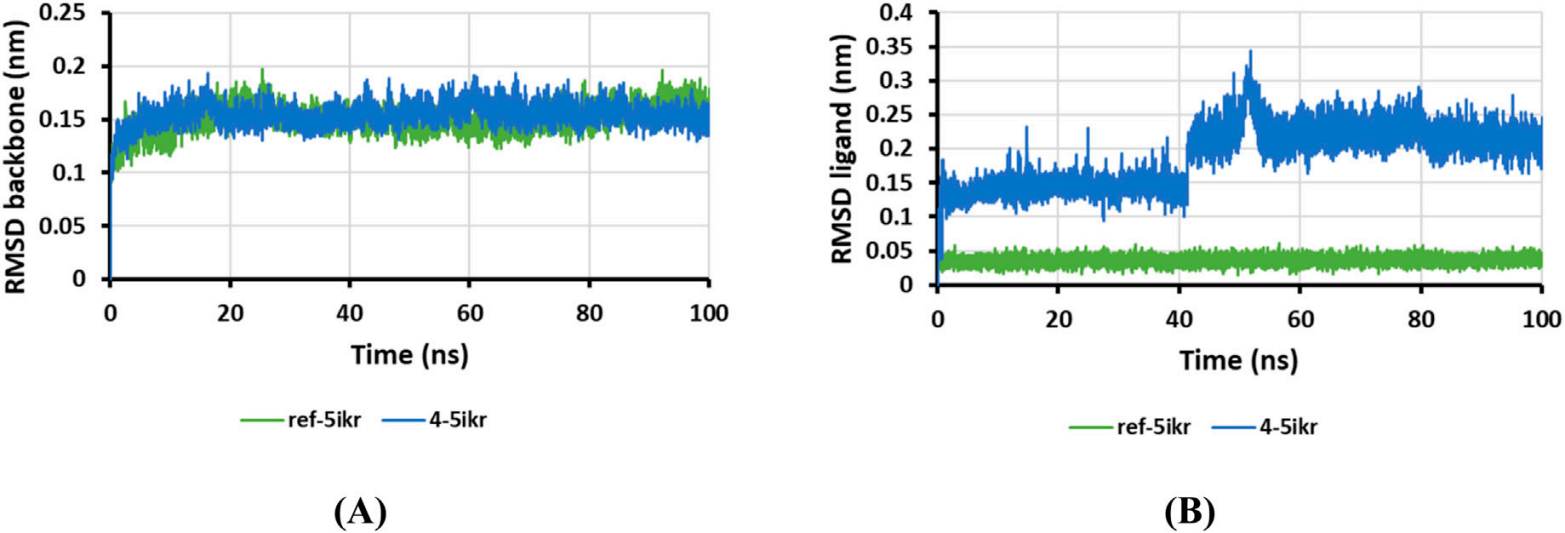
RMSD plot of protein backbone in complexes **(A)** and ligand in complexes **(B)**.

The RMSD plots for the protein backbone and ligand, shown in Figure 7, indicate that the protein backbone exhibited excellent stability in both the 4-COX-2 complex and the reference COX-2 complex (mefenamic acid-COX-2), with average RMSD values of 0.1497 nm and 0.1541 nm, respectively. This demonstrates minimal structural changes in the protein. While compound 4 displayed slight fluctuations between 40 and 50 ns during the simulation, it quickly stabilized and maintained consistency for the remainder of the 100 ns simulation, with an average RMSD of 0.19038 nm. These findings highlight the structural stability of the 4-COX-2 complex and confirm the strong binding affinity of CBSH for COX-2 throughout the simulation.

## 3 Materials and methods

### 3.1 Chemistry

The melting point of the target compound was measured using the Electrothermal IA 9000 instrument without any adjustments. Nuclear magnetic resonance (NMR) spectra, specifically the ^1^H-NMR (400 MHz) and ^13^C-NMR (100 MHz), were recorded using a Bruker spectrometer, with TMS serving as the internal standard. The mass spectrum was carried out on the Direct Inlet part to the mass analyzer in Thermo Scientific GCMS model ISQ. To monitor the reactions, thin-layer chromatography (TLC) was employed, utilizing silica gel and aluminum sheets 60 F254 from Merck. The eluent consisted of a chloroform and methanol mixture in a 9.8:0.2 v/v ratio, and the TLC plates were treated with an iodine-potassium iodide reagent for visualization.

#### 3.1.1 Synthesis of N′-(1-(7-Hydroxy-2-oxo-2H-chromen-3-yl)ethylidene)-4-methylbenzenesulfonohydrazide (CBSH)

In a 25 mL round-bottom flask, a mixture of 3-acetyl-7-hydroxy-2*H*-chromen-2-one (1.0 g, 4.90 mmol) and *p*-toluenesulfonylhydrazide (0.91 g, 4.90 mmol) was dissolved in methanol (15 mL). To this solution, a few drops of hydrochloric acid (1 mL) were added. The mixture was gently warmed until a clear solution was obtained, then allowed to stand at room temperature overnight (12 h). The progress of the reaction was monitored by thin-layer chromatography (TLC). After completion, the resulting precipitate was collected by filtration and washed with cold methanol. The crude product was further purified by recrystallization from acetic acid to afford CBSH as buff-colored crystals. Yield: 1.5 g (82%).

Mp: 226°C–227°C. IR (KBr), ν (cm^−1^): 3326 (ν_OH_), 3217 (ν_NH_ sulfonamid), 1687 (ν_C=O_ lactone), 1614 (ν_C=N_), 1566 (ν_C=C_ arene). ^1^H-NMR (500 MHz, DMSO): δ *ppm*: 2.06 (s, 3H, CH_3_), 2.35 (s, 3H, CH_3_ Ar), 6.69 (s, 1H, H_8_), 6.77 (d, *J* = 8.30 Hz, 1H, H_6_), 7.37 (d, *J* = 9.0 Hz, 2H, H_11_), 7.58 (d, *J* = 8.50 Hz, 1H, H_5_), 7.74 (d, *J* = 8.50 Hz, 2H, H_10_), 7.77 (s,1H, H_4_), 10.54 (s, 1H, NH), 10.73 (brs, 1H, OH). ^13^C-NMR (75 MHz, DMSO): δ *ppm:* 16.44 (C_CH3_), 21.09 (C_CH3_Ar), 101.83 (C8), 111.05 (C4a), 113.71 (C6), 121.48 (C3), 127.57 (C10), 129.61 (C11), 130.82 (C5), 136.16 (C12), 141.79 (C4), 143.49 (C9), 152.21 (C3′), 155.55 (C8a), 159.47 (C2), 162.04 (C7) analysis for C_18_H_16_N_2_O_5_S, M. wt. (372.40), calcd: % C, 58.06; H, 4.33; N, 7.52; found: % C, 58.27; H, 4.55; N, 7.77. MS m/z (R.A.%): 372 (M^+^) (14.64%), 95 (100.00%).

### 3.2 Biological assays

#### 3.2.1 Assessment of antimicrobial activity using the agar cup plate diffusion technique

The antimicrobial efficacy of the newly synthesized compound was assessed utilizing the agar cup plate diffusion method. The study involved the examination of various microbial pathogens, including Methicillin-Resistant *Staphylococcus aureus* (MRSA) ATCC (a Gram-positive bacterium), *Bacillus* cereus ATCC (another Gram-positive bacterium), and Carbapenem-resistant *Pseudomonas aeruginosa* (CRPA) ATCC (a Gram-negative bacterium), in addition to fungal pathogens such as *Candida* albicans ATCC and Aspergillus niger ATCC. Prior to testing, each bacterial pathogen was pre-activated in nutrient broth medium (Condalab, Spain) at 37°C for 24 h, while the fungal pathogens were activated in Potato Dextrose Broth (Condalab, Spain) at 28°C for 48 h under shaking conditions. The inoculum size for each microbial pathogen was determined using the serial dilution method, and the concentration was accurately quantified in terms of Colony Forming Units (CFU) to maintain consistency across all experimental trials. The screening of the compound CBSH against bacterial and fungal pathogens was conducted in accordance with the Clinical and Laboratory Standards Institute (CLSI) protocols (Sroor et al., 2024). A fixed concentration of CBSH (20 µg/mL) was employed alongside standard antibiotic agents. Following the incubation period, the results for both the novel compound and the standard antibacterial agents were evaluated based on the diameter of the inhibition zone (measured in millimeters) (El-Bendary et al., 2021).

To ascertain the Minimum Inhibitory Concentration (MIC) value, CBSH was prepared as a stock solution and subsequently diluted to achieve concentrations ranging from 5 to 100 µg/mL, following the standard broth microdilution method as recommended by CLSI (Singh et al., 2021). The MIC value for each compound was initially determined using the turbidometry method and subsequently confirmed through the CFU method. The MIC for CBSH is defined as the lowest concentration of the compound that results in a reduction in the number of colony-forming units (CFU) when compared to untreated samples (Ragab et al., 2023).

#### 3.2.2 *In vitro* wound scratch assay

The migration rates of human fibroblast (BJ) cells were assessed using the scratch assay method to evaluate the effectiveness of our newly developed compound in enhancing wound healing. We plated 2 × 10^5^ cells in each well of a 24-well plate and cultured them in complete RPMI medium supplemented with 10% fetal bovine serum (FBS), 2 mM L-glutamine, and 1% penicillin-streptomycin, under conditions of 37°C and 5% CO2. After a 24-h incubation, the cells were starved in a medium with 0.5% FBS for another 24 h. The confluent cell layer was then scraped horizontally with a sterile P200 pipette tip to create a wound-like scratch, and debris was removed by rinsing with phosphate-buffered saline (PBS). The cells were treated with CBSH at a low concentration of 12.5 µg/mL, while untreated cells acted as a negative control. The initial scratch, representing the wound, was photographed at 0 h using phase contrast microscopy at ×4 magnification before the CBSH treatment. After another 24-h incubation, a second set of images was taken. The migration rate was evaluated by analyzing the images with ImageJ software, measuring the percentage of area closure, and comparing it to the value at 0 h. An increase in the percentage of closed areas indicated cell migration and the potential of CBSH to aid in wound healing. All experiments were performed in triplicate, and the data were calculated according to the specified formula.

$$\text{Wound closure }\left(\%\right)=\left(\text{Measurement at }0\;h-\text{Measurement at }24\;h\right)\;/\text{ Measurement at }0\;h\times 100$$

#### 3.2.3 Development of *in vivo* models for wound healing assessment

In the present investigation, a cohort of ten healthy male Wistar Albino rats, each weighing between 180 and 200 g, was randomly divided into two distinct groups. The control group, consisting of five rats, received no treatment, whereas the experimental group, also comprising five rats, was administered CBSH, which was prepared in sterile water at a concentration of 25 mg/mL. A volume of 200 µL of this suspension was applied to the wounds on specified days: 0, 3, 5, 7, 10, and 14 following the creation of the wounds. The rats were obtained from the animal house facility at the Faculty of Pharmacy, Egyptian-Russian University, located in Cairo, Egypt. The *in vivo* experiments were conducted in compliance with the ethical approval number (ERUFP-PC-24-005) issued by the Egyptian-Russian University Research Ethics Committee. Prior to the commencement of the experiment, the rats underwent a 10-day acclimatization period and were housed under controlled environmental conditions, which included regulated humidity and temperature, as well as a 12-h light/dark cycle.

In the process of wound creation, rats were anesthetized with isoflurane, and the dorsal fur was removed using an electric razor. The sites of the wounds were then disinfected with 70% ethanol, followed by the creation of full-thickness skin wounds utilizing a 10 mm biopsy punch. Immediate photographic documentation of the wounds was conducted with a digital camera. Subsequent images of the wound area were captured over a two-week period on days 0, 3, 5, 7, 10, and 14 to facilitate monitoring of the healing process. On the 14th day, the wounds were excised under anesthesia for histopathological analysis. The quantification of the wound area was performed using open-source ImageJ software, which enabled the calculation of the wound area by establishing a digital correlation between the image pixels and their corresponding measurements in millimeters. Ultimately, the wound area was determined based on these quantitative assessments.

#### 3.2.4 Histopathology of wound healing

At the conclusion of the experiment, the dissected wound and the adjacent tissue were rinsed with normal saline and subsequently immersed in 10% neutral buffered formalin. The samples were fixed for a duration of 24 h before being processed for paraffin embedding. Initially, the samples underwent dehydration using ethyl alcohol in ascending concentrations, followed by clearing in xylene. They were then impregnated with soft paraffin and subsequently embedded and blocked in hard paraffin. Utilizing a rotary microtome, the paraffin blocks were sectioned to a thickness of 5 µm. The resulting sections were mounted on clean, dry glass slides and stained with Haematoxylin and Eosin (H&E) as well as Masson’s trichrome stain. Finally, these stained sections were examined using a LEICA DFC290 HD system digital camera, connected to a light microscope, employing 10X, 20X, and 40X objective lenses (Bancr et al., 2008). The histopathological examination was conducted in the histopathology laboratory within the Department of Histology, Faculty of Veterinary Medicine, Beni-Suef University.

### 3.3 Molecular modeling and molecular dynamics simulation

The chemical structures of CBSH were drawn using Marvin JS software and then energy-optimized through the MMFF94s force field using Avogadro software (Halgren, 1999). The crystal structures of the proteins tumor necrosis factor-alpha (TNF-α, PDB code: 2AZ5), arachidonate 5-lipoxygenase (ALOX5, PDB code: 6N2W), and cyclooxygenases (COX-1, PDB code: 6Y3C; COX-2, PDB code: 5IKR) were downloaded from the RCSB Protein Data Bank and processed for molecular docking simulations in a manner similar to previous studies (Giang et al., 2025). In our computational workflow, a grid box was centered on the co-crystallized ligand to encompass the binding site of each protein. Default parameters were used, except for the exhaustiveness, which was set to 400 to enhance precision. Molecular docking simulations were conducted using AutoDock Vina v1.2.3 (Trott and Olson, 2010; Eberhardt et al., 2021). To analyze and visualize the mutual interactions between the proteins and the studied ligands, BIOVIA Discovery Studio Visualizer software was employed, offering clear insights into the molecular binding modes. Furthermore, molecular dynamics simulations were conducted using GROMACS 2023.3 with the setup protocol as described in a previous report (Makarem et al., 2019).

## Data Availability

The original contributions presented in the study are included in the article/Supplementary Material, further inquiries can be directed to the corresponding authors.
