# Supplementary material for: Discovery of N′-(1-(coumarin-3-yl)ethylidene)benzenesulfonohydrazide as a novel wound healing enhancer: synthesis, biological assessment, and molecular modeling
Source: Front Chem. 2025 Aug 8;13:1621717. doi: 10.3389/fchem.2025.1621717 (PMC12370739; doi:10.3389/fchem.2025.1621717)
Supplement: Supplementary file 1 [file DataSheet1.pdf]

Supporting Information

Discovery of *N'*-(1-(coumarin-3-yl)ethylidene)benzenesulfonohydrazide as a novel wound healing enhancer: Synthesis, biological assessment, and molecular modeling

Eman F. Khaleel [a], Heba Abdelmegeed [b], Abdel-Razik H. Abdel-Razik [c], Manal S Ebaid [d], Ninh The Son [e], Nguyen Xuan Ha [f], Hoda Atef Abdelsattar Ibrahim [g], Mohamed A. Abdelrahman [h,i], Abdelsamed I. Elshamy [b], Jarosław Dziadek [j*], Ahmed Sabt [b], Wagdy M. Eldehna [k,*]

[a] *Department of Medical Physiology, College of Medicine, King Khalid University, Asir 61421, Saudi Arabia*
[b] *Chemistry of Natural Compounds Department, Pharmaceutical and Drug Industries Research Institute, National Research Center, Dokki, Cairo 12622, Egypt*
[c] *Department of Histology, Faculty of Veterinary Medicine, Beni-Suef University, Beni-Suef 62511, Egypt*
[d] *Department of Chemistry, College of Science, Northern Border University, Arar, Saudi Arabia*
[e] *Institute of Chemistry, Vietnam Academy of Science and Technology (VAST), 18 Hoang Quoc Viet, Caugiay, Hanoi 10000, Viet Nam*
[f] *Institute of Natural Products Chemistry, VAST, 18 Hoang Quoc Viet, Caugiay, Hanoi 10000, Viet Nam*
[g] *Pediatric Department, Faculty of Medicine, Cairo University, Cairo, Egypt*
[h] *Department of Pharmacy, Kut University College, Al Kut, Wasit, 52001, Iraq*
[i] *Department of Pharmaceutical Chemistry, Faculty of Pharmacy, Egyptian Russian University, Badr City, Cairo, Egypt*
[j] *Laboratory of Genetics and Physiology of Mycobacterium, Institute of Medical Biology of the Polish Academy of Sciences, Lodz, Poland.*
[k] *Department of Pharmaceutical Chemistry, Faculty of Pharmacy, Kafrelsheikh University, Kafrelsheikh, P.O. Box 33516, Egypt.*

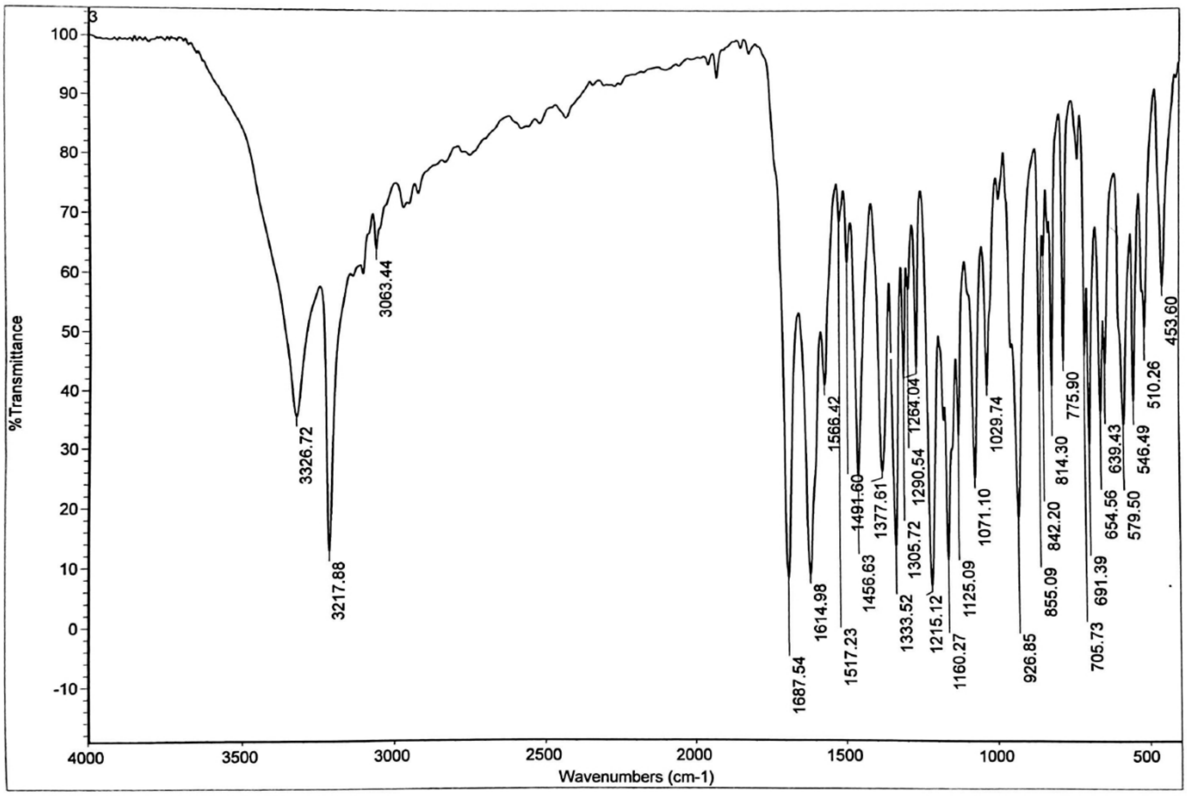

Figure S1: IR spectrum of compound CBSH.

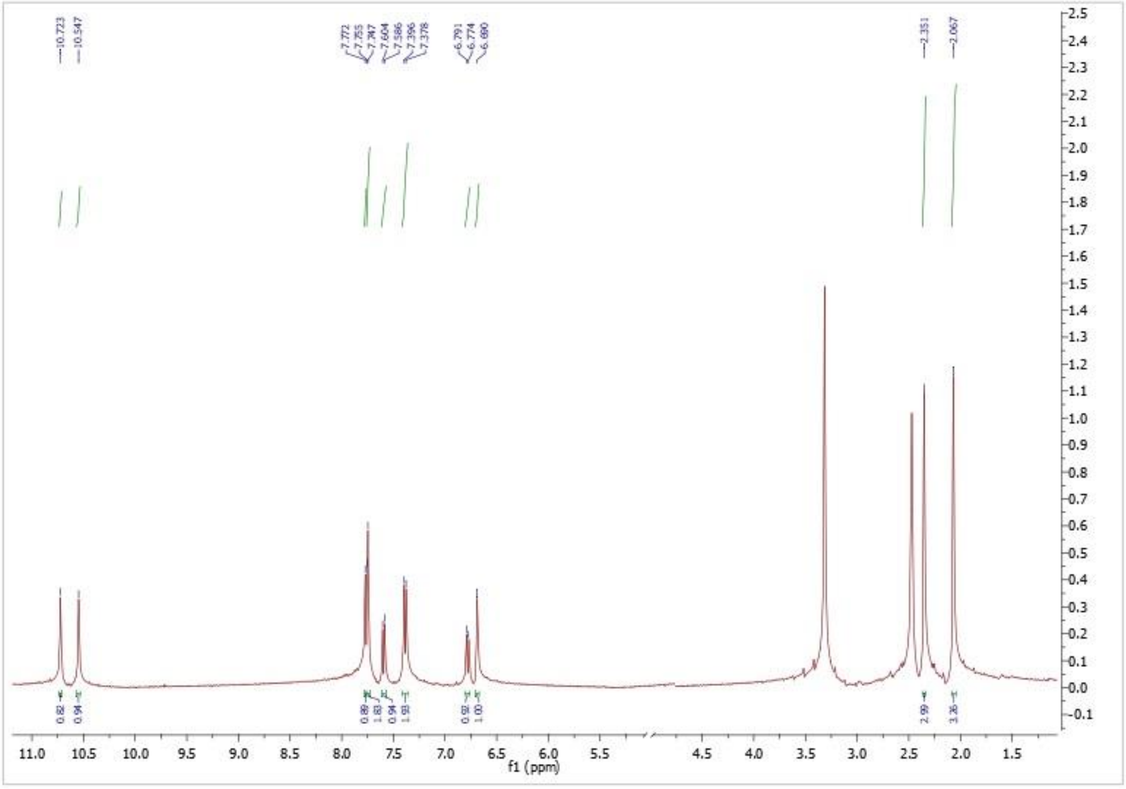

Figure S2: ^{1}H-NMR (300 Hz, DMSO-d_6) of compound CBSH.

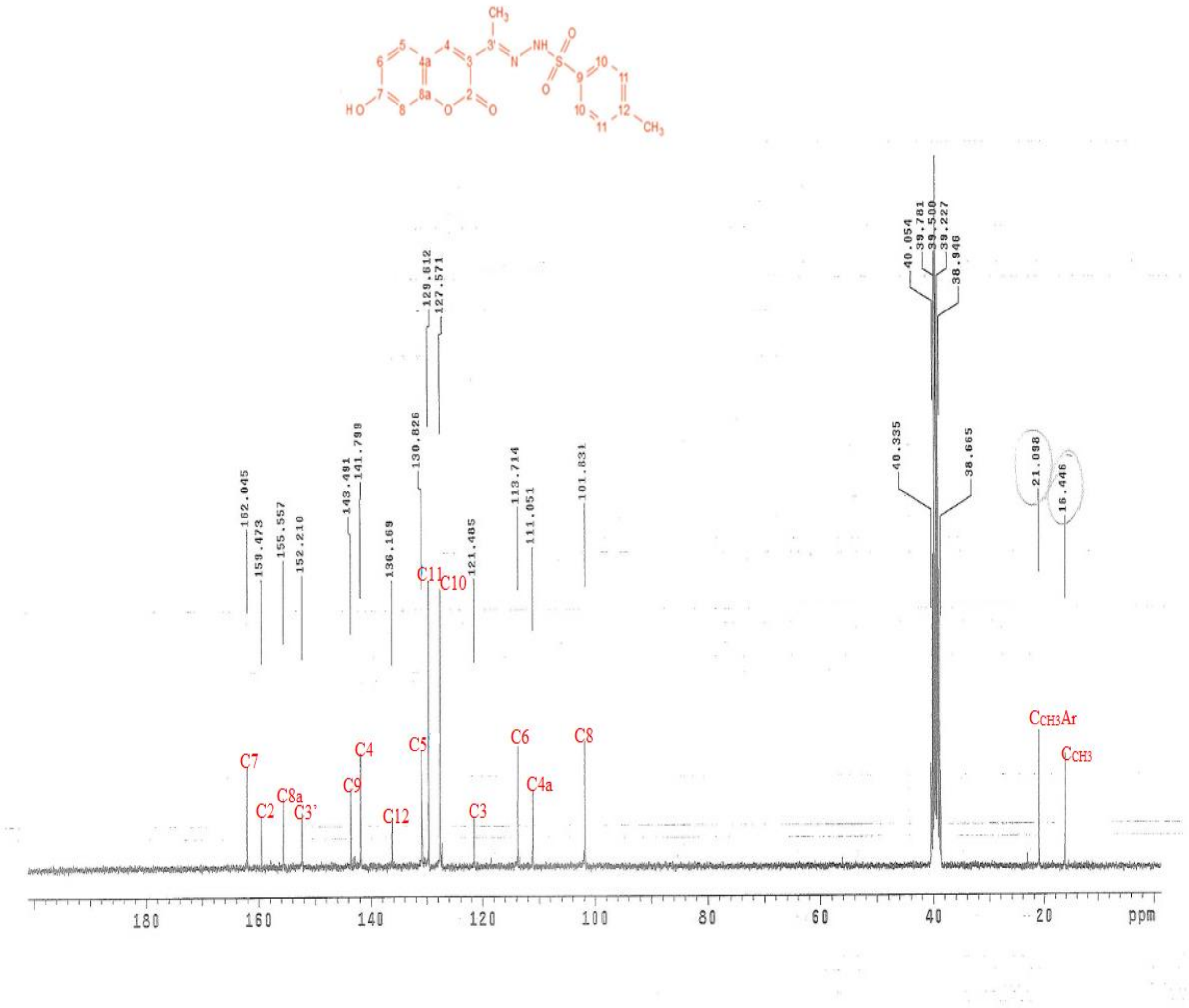

Figure S3: ^{13}C-NMR (75 MHz, DMSO-d_6) of compound CBSH

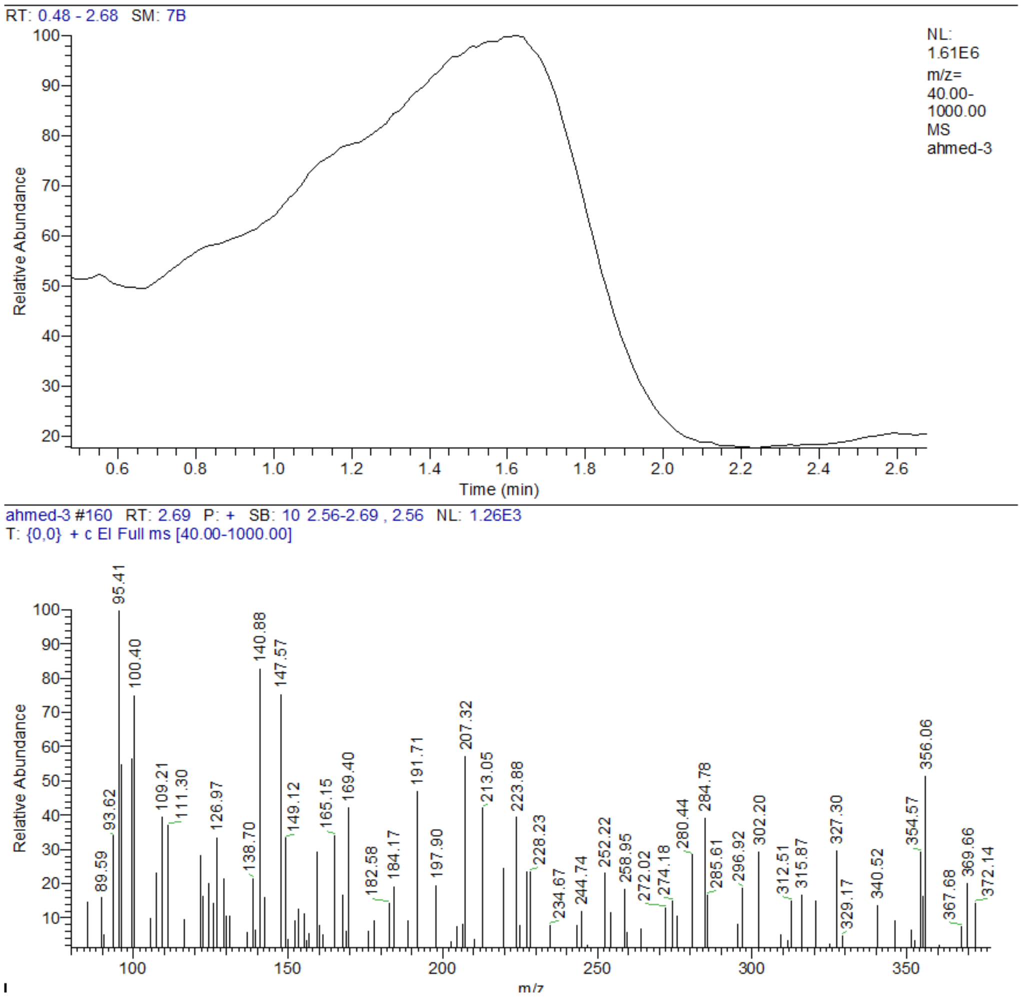

Figure S4: mass spectroscopic of compound CBSH

***Staphylococcus aureus* MRSA**

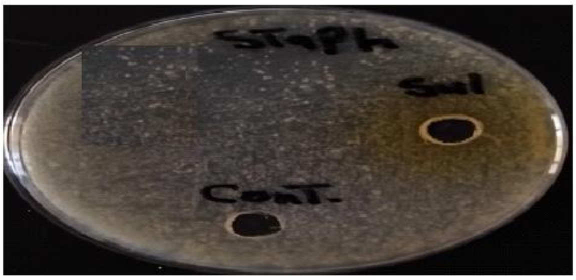

Pseudomonas aeruginosa

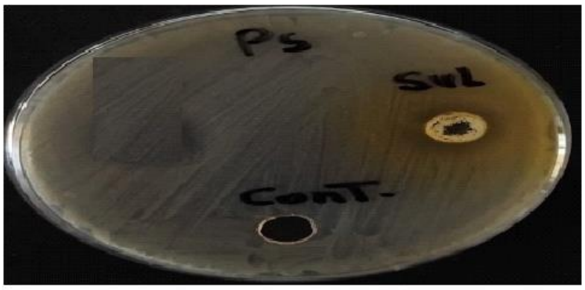

Bacillus cereus

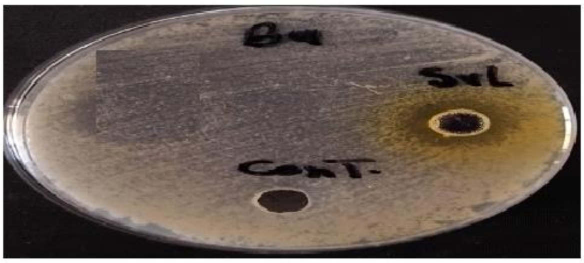

Candida albicans

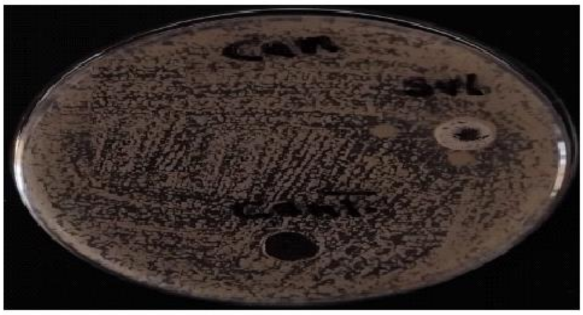

Aspergillus niger

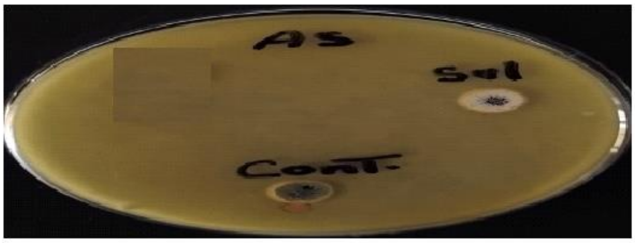

Figure S5. Antibacterial activity of the targeted compound CBSH and standard control using agar-well diffusion

***Staphylococcus aureus* MRSA**	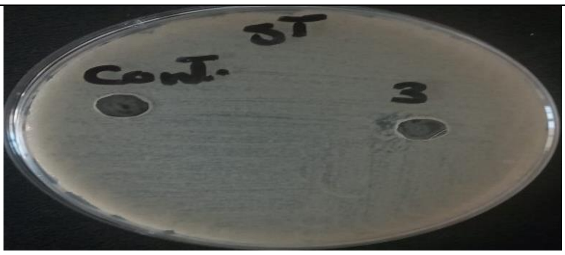
Pseudomonas aeruginosa	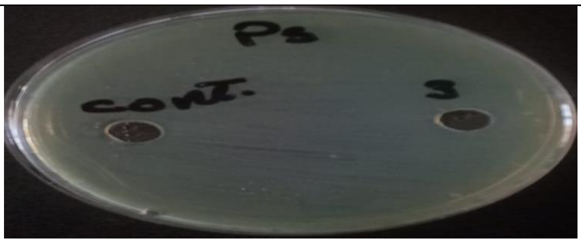
Bacillus cereus	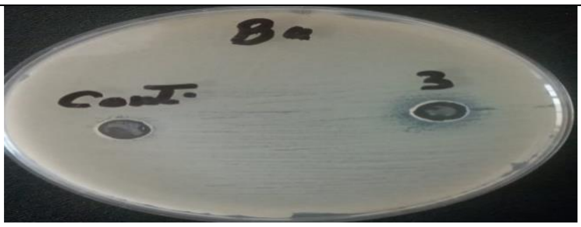
Candida albicans	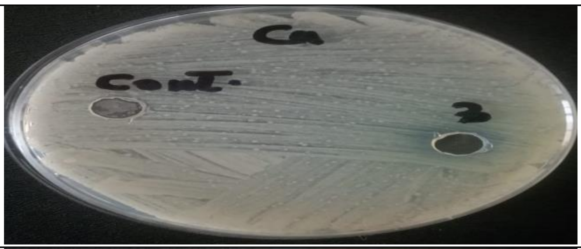
Aspergillus niger	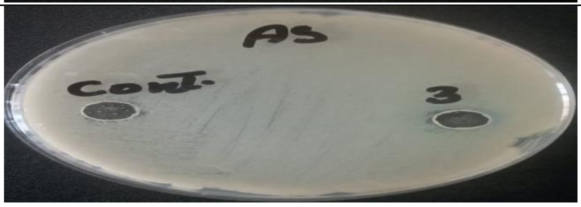

Figure S6. Antibacterial activity of the compound 3 using agar-well diffusion

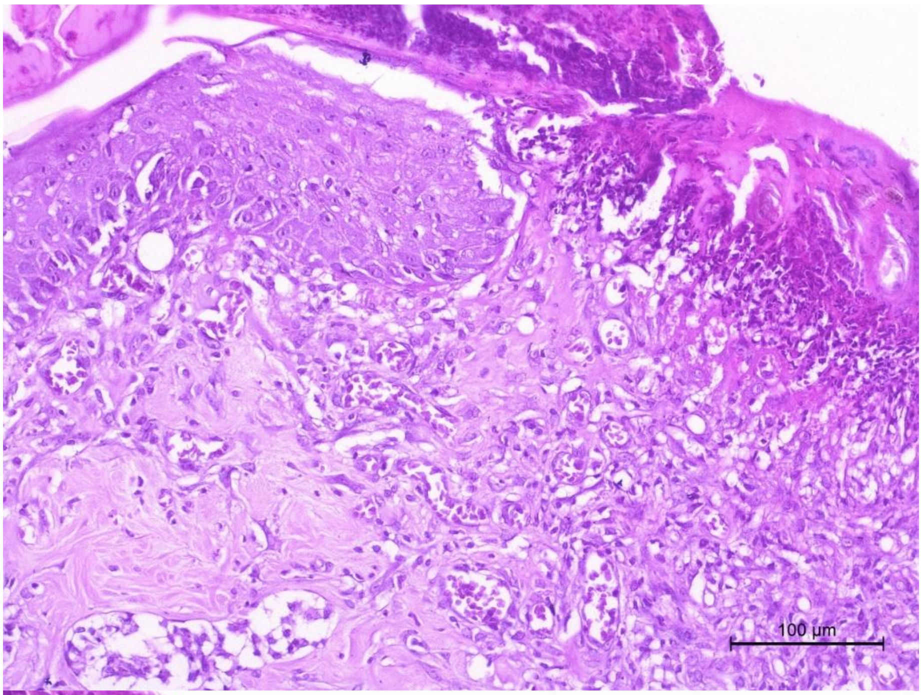
100 µm

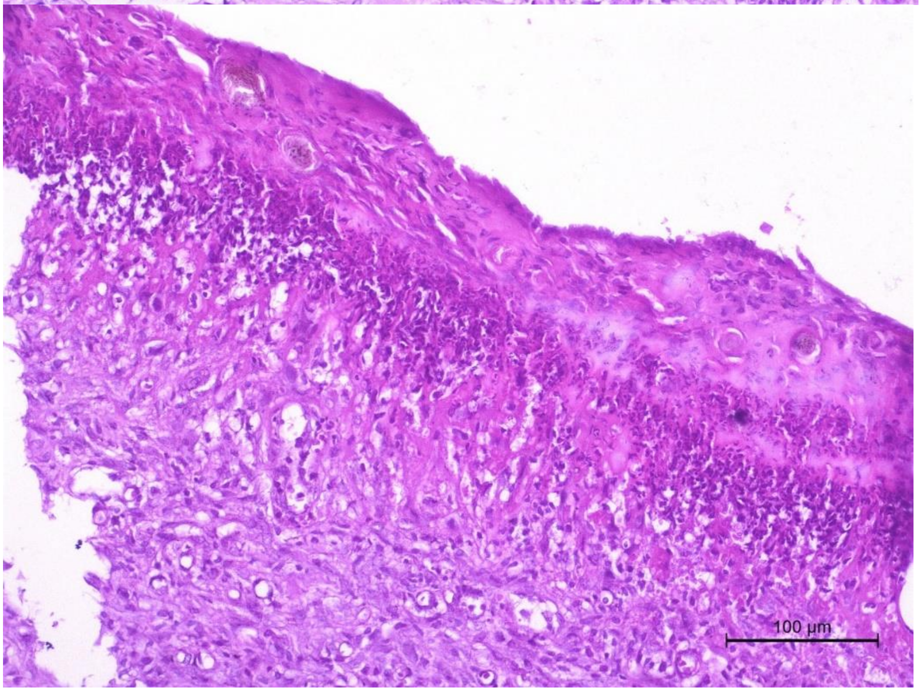
100 µm

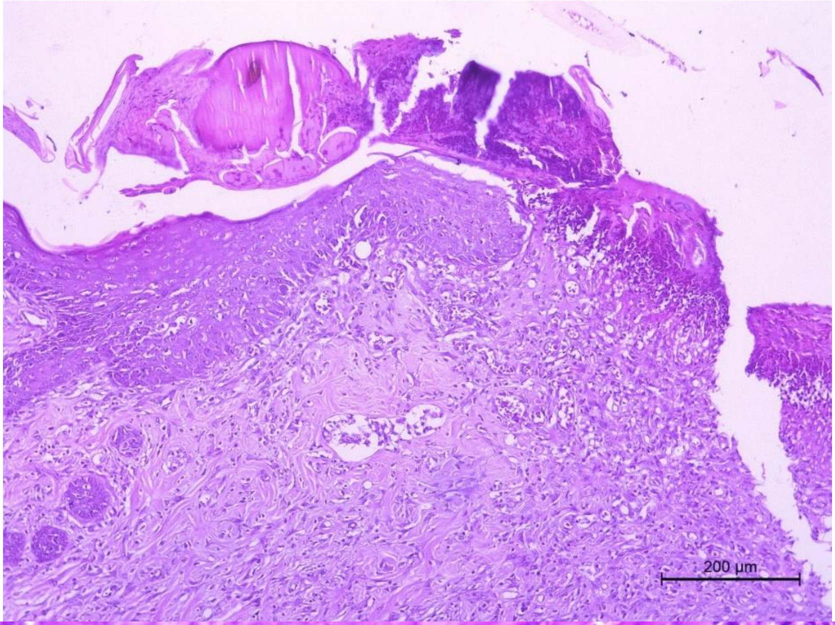
200 µm

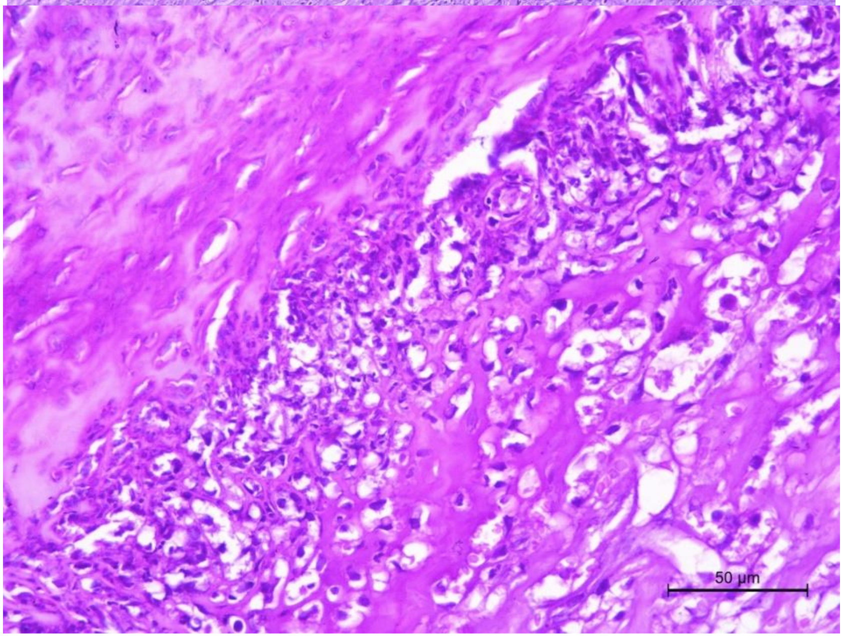
50 µm

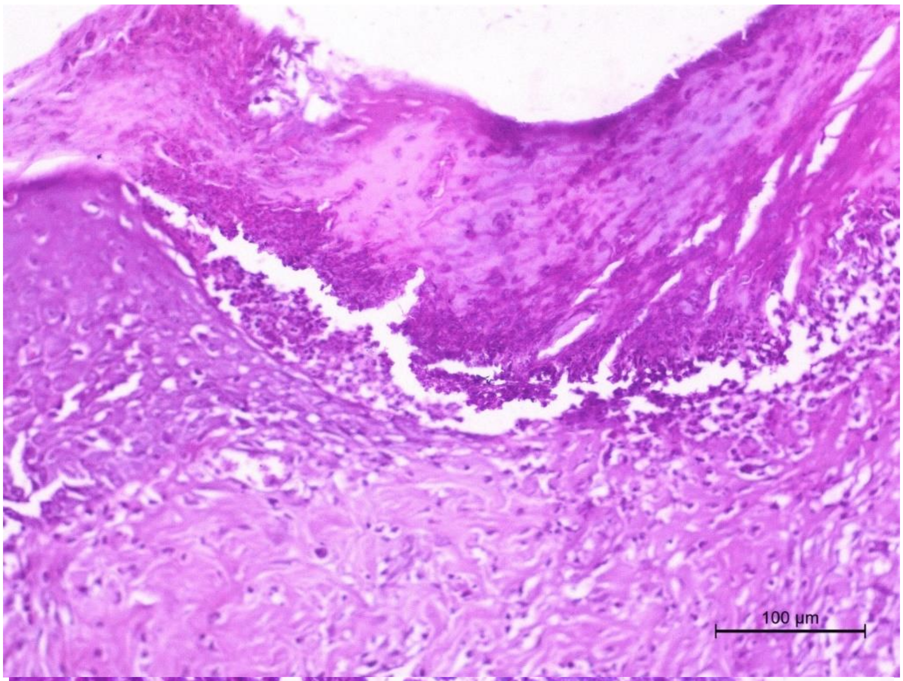
100 µm

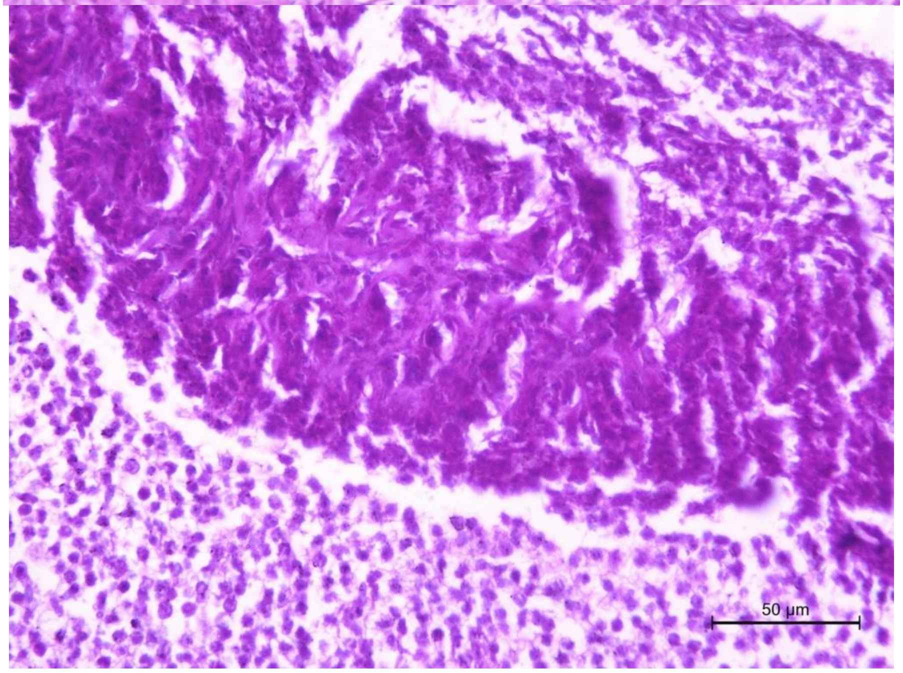
50 µm

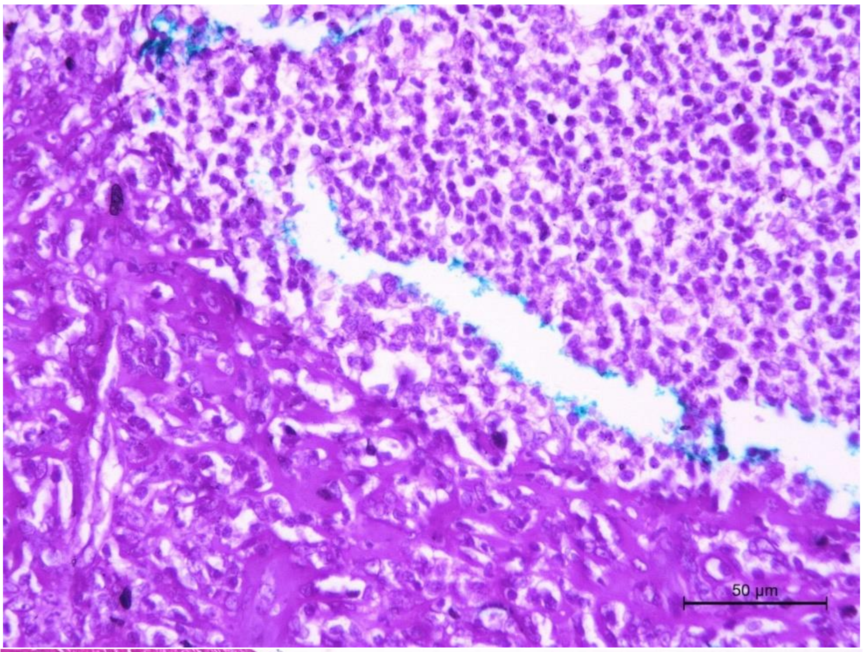
50 µm

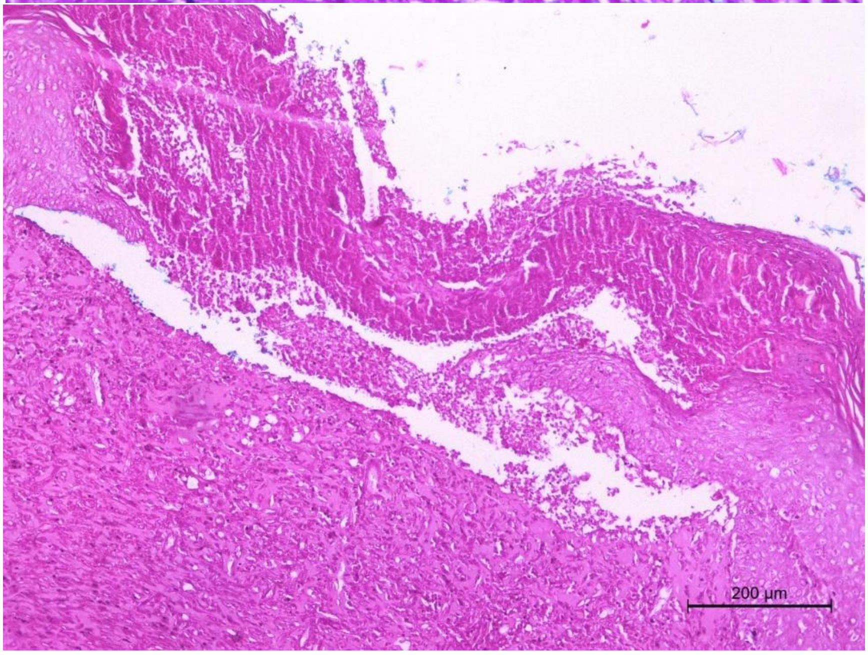
200 µm

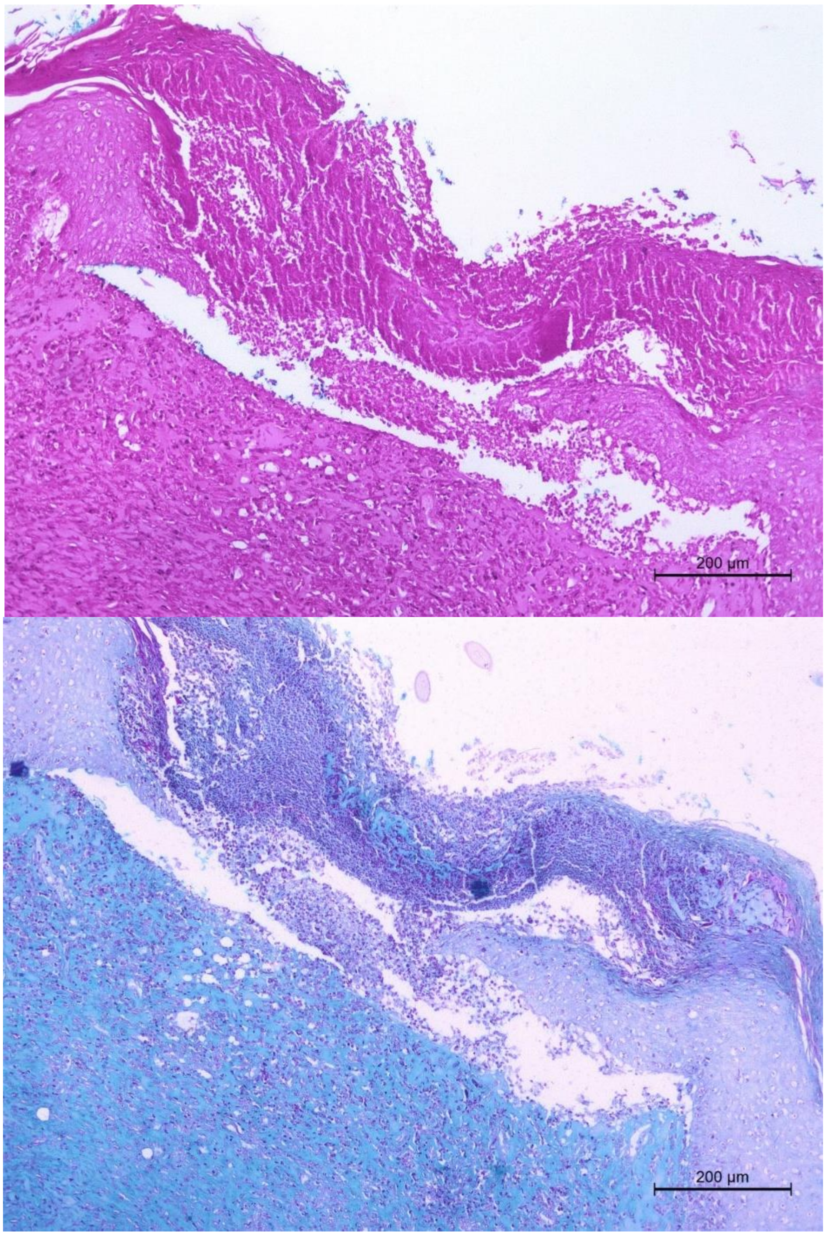

Figure S7: The histopathological picture for the control positive group.

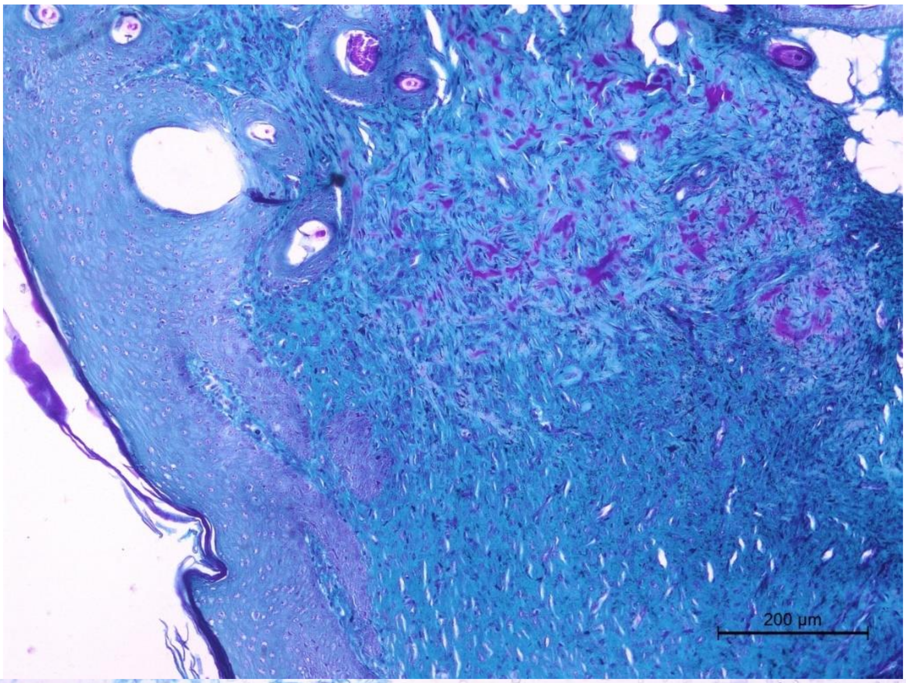
200 µm

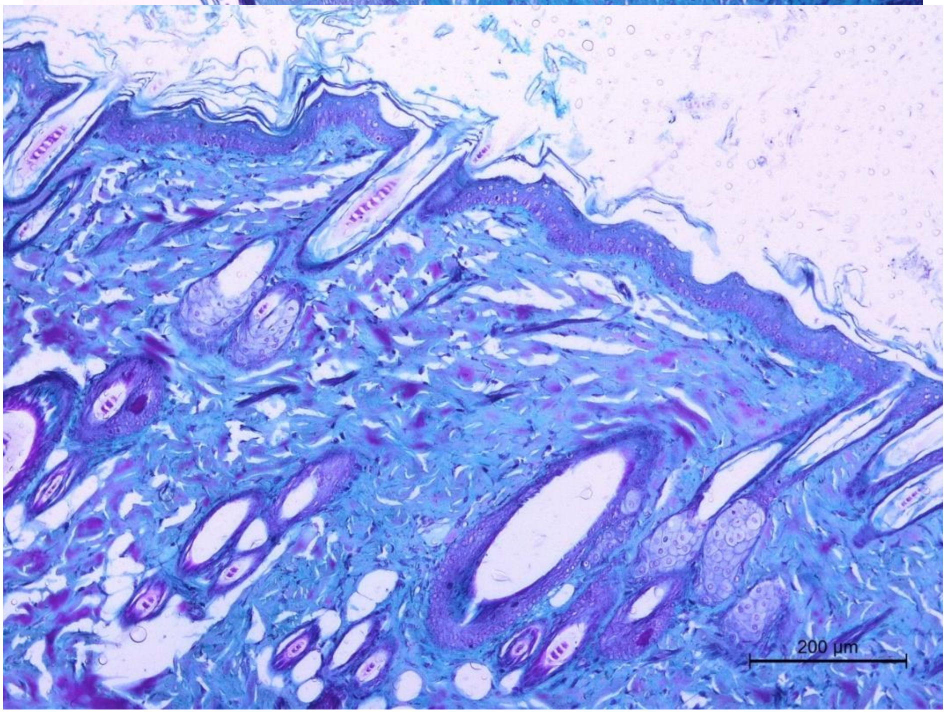
200 µm

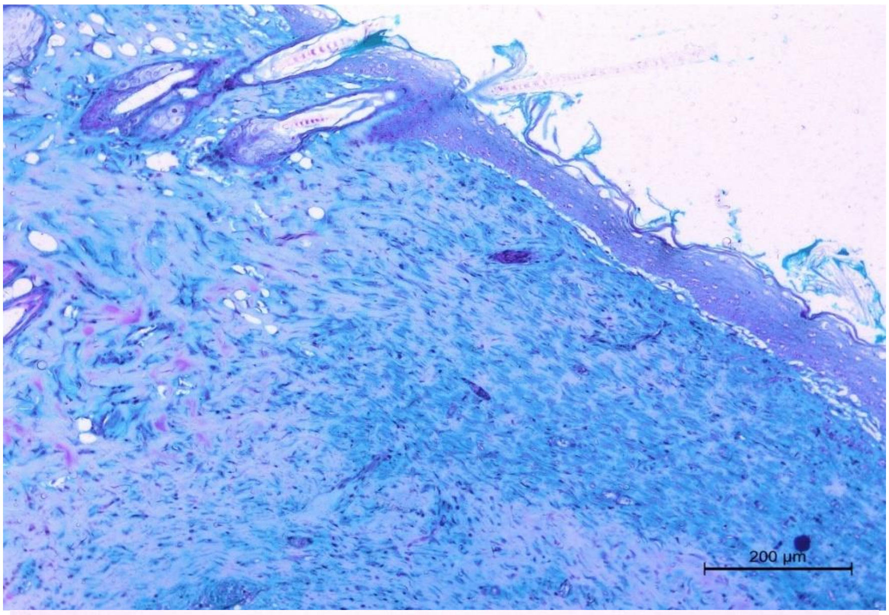
200 µm
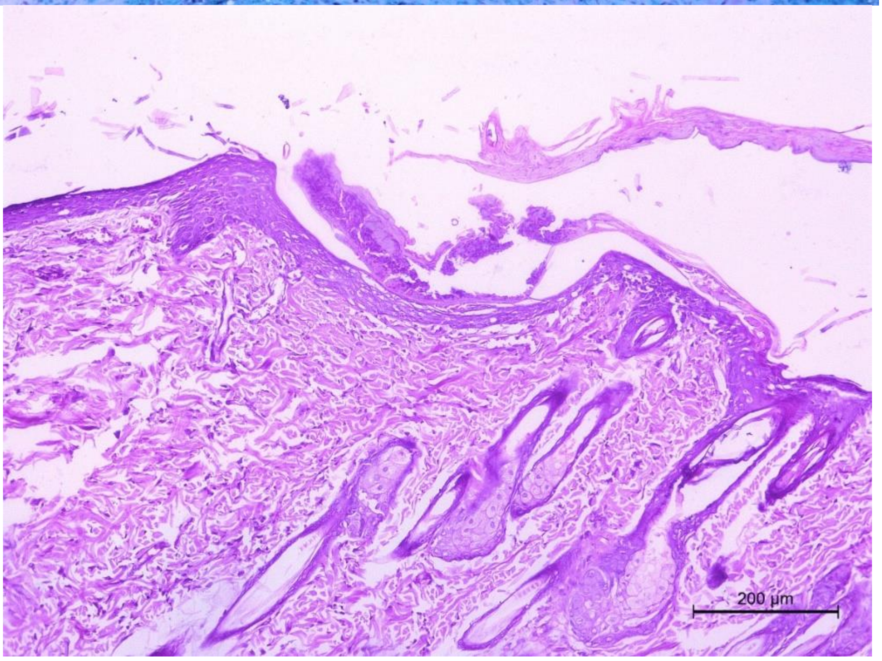
200 µm

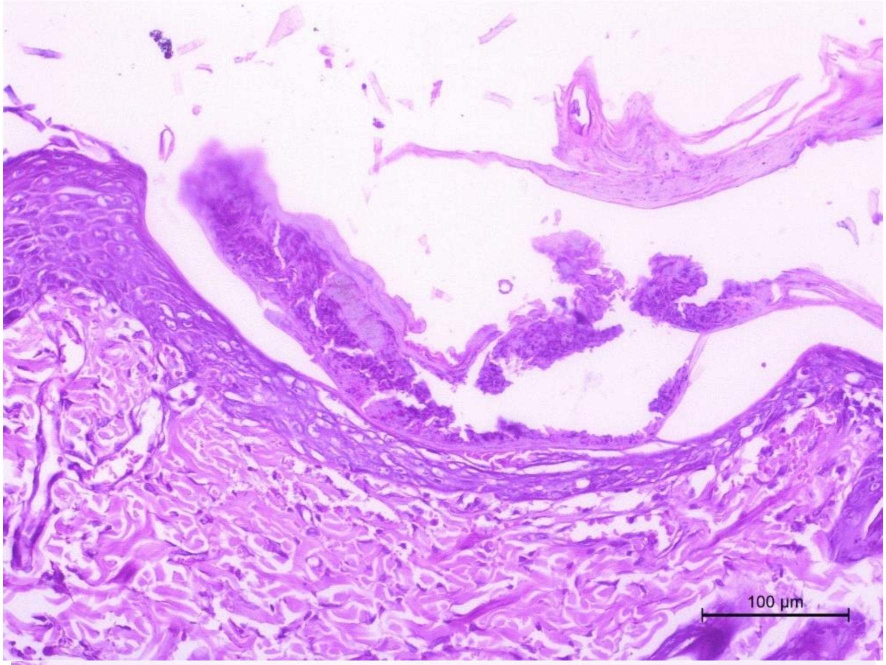
100 µm

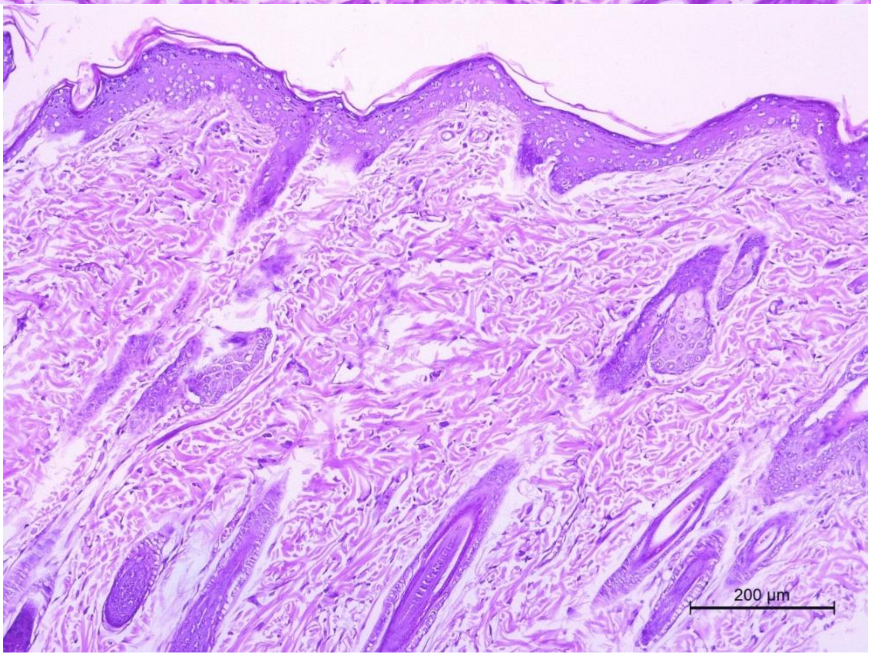
200 µm

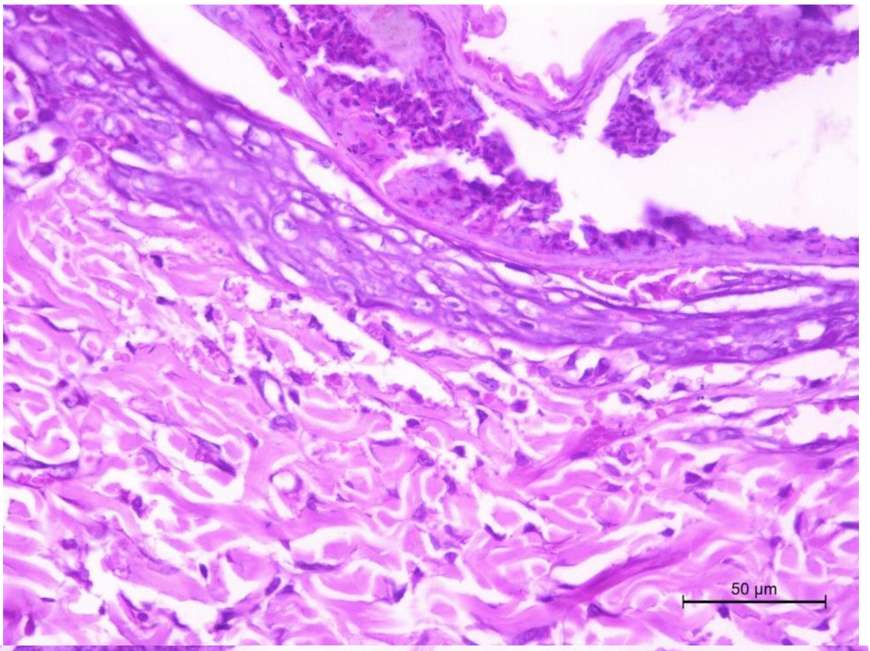
50 μm

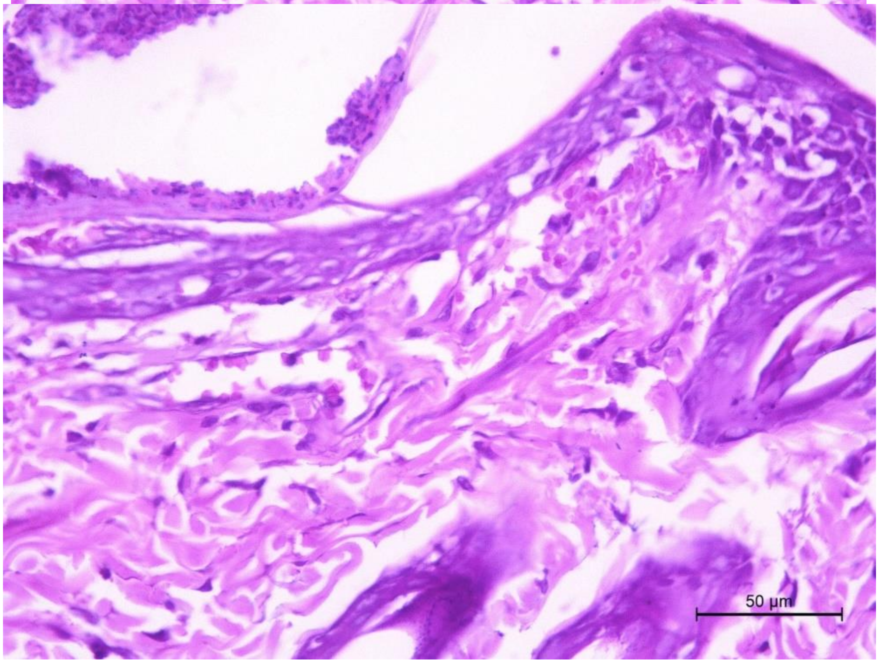
50 μm

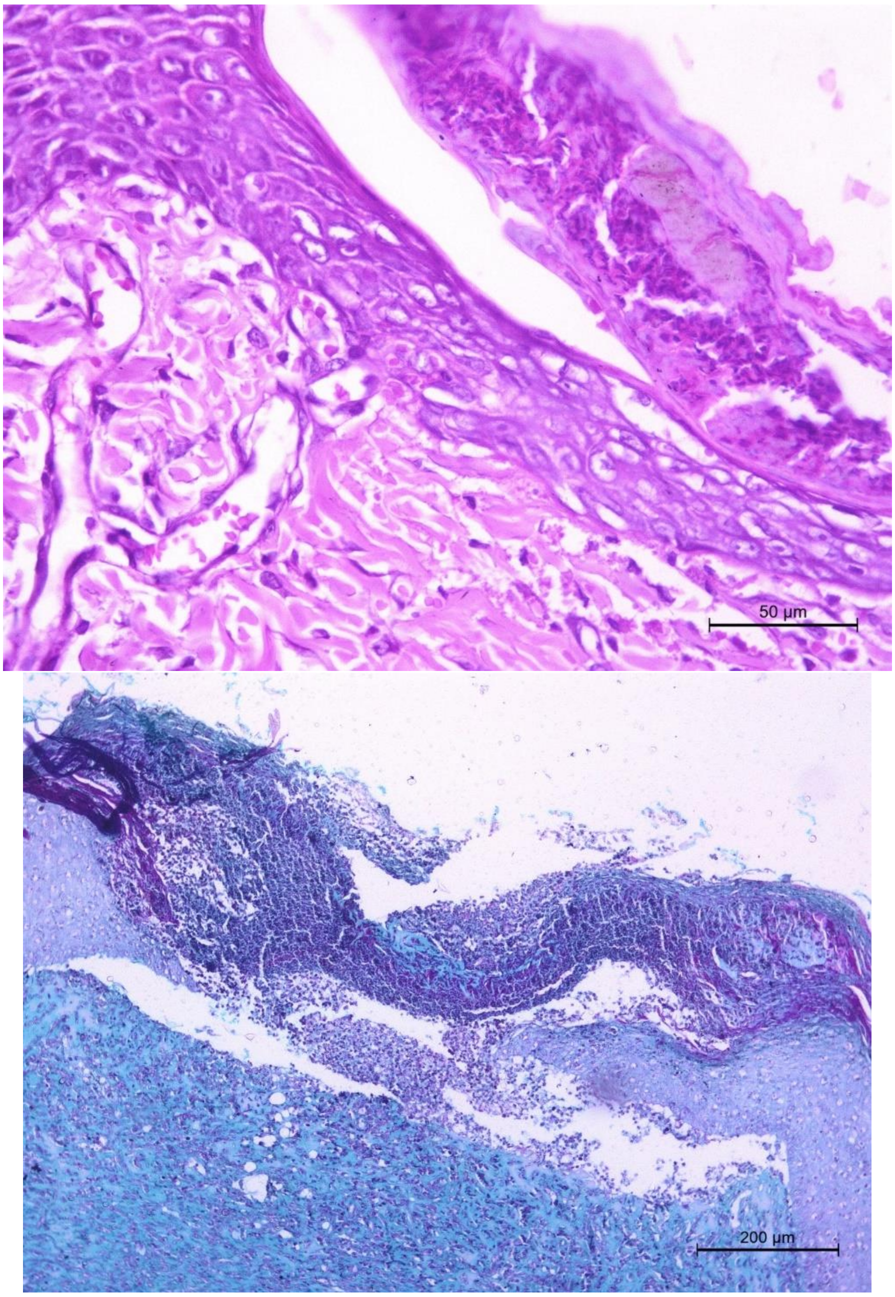

Figure S8. The histopathological picture of **CBSH**.

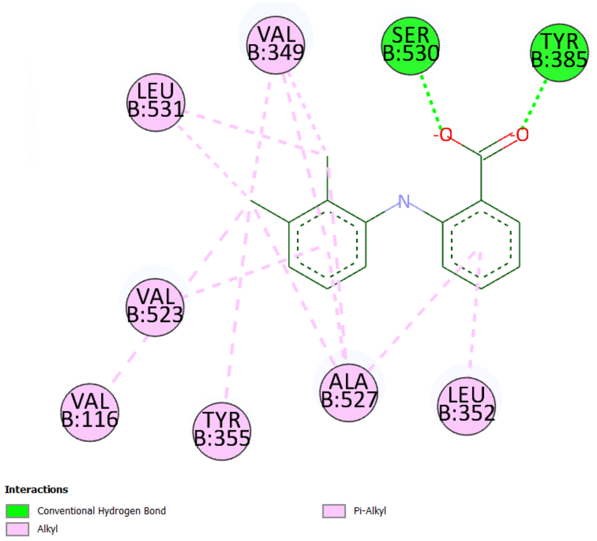

Figure S9. 2D interaction view of Mefenamic within the active site of COX-2 (PDB code: 5IKR).
